# Exploration of the mechanism of Traditional Chinese Medicine for anxiety and depression in patients with diarrheal irritable bowel syndrome based on network pharmacology and meta-analysis

**DOI:** 10.3389/fphar.2024.1404738

**Published:** 2024-05-21

**Authors:** Chen Bai, Junyi Wang, Yifan Wang, Haoqi Liu, Jiaxiu Li, Siyi Wang, Zhen Bai, Rongjuan Guo

**Affiliations:** ^1^ Dongfang Hospital, Beijing University of Chinese Medicine, Beijing, China; ^2^ Department of Medical Equipment, The First Affiliated Hospital of Zhengzhou University, Zhengzhou, China; ^3^ Psychosomatic Department, Dongfang Hospital, Beijing University of Chinese Medicine, Beijing, China

**Keywords:** diarrheal irritable bowel syndrome, anxiety, depression, Chinese herbal medicine, meta-analysis

## Abstract

**Background:**

The efficacy of Chinese herbal medicine (CHM) in managing irritable bowel syndrome with diarrhea (IBS-D) accompanied by anxiety and depression remains uncertain. Thus, a systematic review was carried out employing meta-analysis and network pharmacology to ascertain the efficacy and underlying mechanisms of CHM therapy.

**Methods:**

By conducting a systematic review, including literature search, screening, and data extraction, we identified 25 randomized controlled trials to assess CHM’s effectiveness in treating irritable bowel syndrome alongside anxiety and depression. Network pharmacology was utilized to scrutinize the metabolite utility of CHM in addressing this condition. Potential primary mechanisms were synthesized using information sourced from the PubMed database.

**Results:**

Twenty-five studies, including 2055 patients, were analyzed, revealing significant treatment efficacy for IBS-D in the trial group compared to controls [OR = 4.01, 95% CI (2.99, 5.36), I^2^ = 0%] Additionally, treatment for depression [SMD = −1.08, 95% CI (-1.30, −0.86), *p* < 0.00001, I^2^ = 68%; SDS: SMD = -1.69, 95% CI (-2.48, −0.90), *p* < 0.0001, I^2^ = 96%] and anxiety [HAMA: SMD = -1.29, 95% CI (-1.68, −0.91), *p* < 0.00001, I^2^ = 89%; SAS: SMD = -1.75, 95% CI (-2.55, −0.95), *p* < 0.00001, I^2^ = 96%] significantly improved in the trial group. Furthermore, the trial group exhibited a significantly lower disease relapse rate [OR = 0.30, 95% CI (0.20, 0.44), *p* < 0.00001, I^2^ = 0%]. CHM treatment consistently improved IBS severity (IBS-SSS) and symptom scores. Network pharmacology analysis identified key chemical metabolites in traditional Chinese medicine formulations, including Beta-sitosterol, Stigmasterol, Quercetin, Naringenin, Luteolin, Kaempferol, Nobiletin, Wogonin, Formononetin, and Isorhamnetin. Utilizing the STRING database and Cytoscape v3.9.0 software, a protein-protein interaction (PPI) network revealed the top eight key targets: IL-6, TNF, PPARG, PTGS2, ESR1, NOS3, MAPK8, and AKT1, implicated in anti-inflammatory responses, antioxidant stress modulation, and neurotransmitter homeostasis maintenance.

**Conclusion:**

Chinese Herbal Medicine (CHM) offers a promising and safe treatment approach for patients dealing with Diarrheal Irritable Bowel Syndrome (IBS-D) accompanied by anxiety and depression; thus, indicating its potential for practical implementation. The most active metabolites of CHM could simultaneously act on the pathological targets of IBS-D, anxiety, and depression.The diverse scope of CHM’s therapeutic role includes various aspects and objectives, underscoring its potential for broad utilization.

## 1 Introduction

Abdominal pain or discomfort and alterations in defecation patterns are prevalent symptoms of Irritable Bowel Syndrome (IBS), a common functional gastrointestinal disorder. IBS can be categorized into diarrhea-type, constipation-type, mixed-type, and indeterminate-type based on abnormal defecation patterns (Drossman, 2016). Its etiology is complex, involving visceral hypersensitivity, abnormal gastrointestinal motility, and psychological stress (Ford et al., 2017). Many individuals with IBS-D often encounter psychological symptoms alongside gastrointestinal ones. Growing evidence highlights the strong link between IBS-D and anxiety as well as depression (Chen et al., 2022). The prevalence of anxiety symptoms among IBS patients is estimated at 1.28%, while that of depression symptoms is 8.11% (Zamani et al., 2019). Notably, individuals with IBS-D are three times more likely to experience anxiety or depression compared to healthy individuals (Kurokawa et al., 2018). Anxiety and depressive symptoms may exacerbate gastrointestinal and extra-gastrointestinal symptoms by altering visceral hypersensitivity and the intestinal microenvironment, influencing the microbiota-intestinal-brain axis (Moloney et al., 2016; Ford et al., 2017). Moreover, psychological factors have the potential to disrupt intestinal mucosal integrity, modify gut microbiota composition, impair mucosal barrier function, and modulate immune responses. Taken together, these factors play a collective role in the manifestation of symptoms such as diarrhea in individuals with IBS-D, thereby complicating the management of IBS-D patients. Currently, Western medicine lacks specific medication for treating IBS-D. Clinical management typically involves symptomatic supportive treatment, including antispasmodics, antidiarrheals, antibiotics, anxiolytics, and probiotics, alongside dietary adjustments and psychological interventions.

In modern medicine, IBS-D is classified based on its clinical manifestations. However, in traditional Chinese medicine, it is categorized into broader categories such as “diarrhea,” and “abdominal pain.” Traditional Chinese Medicine (TCM) provides a comprehensive understanding of IBS-D, emphasizing personalized treatment tailored to the individual’s constitution, environment, and specific symptoms. Treatment approaches in TCM are varied, including internal administration of herbal medicine, acupuncture, tuina massage, acupoint patches, and herbal enemas. TCM views IBS-D as primarily affecting the small and large intestines, intricately linked with the liver, spleen, and kidney. Core pathogenesis involves spleen-stomach weakness and liver dysfunction in dispersing and regulating. In clinical practice, liver qi stagnation and spleen deficiency with dampness accumulation are commonly observed syndromes in IBS-D. Herbal medicine treatment adjusts medications based on different syndrome patterns to alleviate symptoms such as diarrhea, abdominal distention, and pain. The mechanism of TCM treatment of IBS-D is multifaceted, including the regulation of intestinal function, neuroendocrine, immune system and other pathways. Through multi-target and multi-mechanism regulation, patients’ diarrhea, abdominal pain, and emotional symptoms can be improved to achieve the purpose of treatment. Most studies suggest that CHM treatments for IBS-D yield better outcomes compared to Western medicine. However, many research reports on CHM treatment of IBS-D have limitations, such as small sample sizes and inconsistencies in clinical efficacy evaluation standards. The clinical efficacy of CHM in managing IBS-D with depression and anxiety requires clarification through influential research. Therefore, this paper comprehensively analyzes published research, conducts meta-analysis and systematic review, and provides a rational, evidence-based medical foundation for exploring the efficacy and mechanisms of CHM in treating IBS-D with depression and anxiety.

## 2 Methods

### 2.1 Literature search strategy

Search terms for the databases specified included “irritable bowel syndrome or IBS-D″ and “anxiety or anxiety disorder” and “depression or depressive disorder” and “Chinese medicine or herbal medicine” and “randomized controlled trial,” along with their synonyms. The databases to be searched included China National Knowledge Infrastructure (CNKI), Wanfang Database, VIP Database, Chinese Biomedical Literature Database, PubMed, Embase, and Cochrane Library, up to 10 April 2024. Both Chinese and English will be used for retrieval.

### 2.2 Inclusion and exclusion criteria

#### 2.2.1 Study inclusion criteria

1) All participants met the diagnostic criteria for IBS-D (Longstreth et al., 2006; Mearin et al., 2016) with comorbid anxiety and depression. 2) Each group included no fewer than 30 subjects. 3) The trial group received oral CHM alone or CHM in combination with Western Medicine, while the control group received Western Medicine excluding Chinese medicine (multiple interventions allowed). 4) Assessment of anxiety and depressive symptoms in patients utilized the Hamilton Depression Scale (HAMD) or Self-rating Depression Scale (SDS), as well as the Hamilton Anxiety Scale (HAMA) or Self-rating Anxiety Scale (SAS). 5) Efficacy indicators included ① Severity of irritable bowel syndrome (IBS-SSS) score, ② TCM symptom score, ③ Clinical efficacy, ④ Recurrence rate, ⑤ Adverse effects.

#### 2.2.2 Study exclusion criteria

1) Non-clinical investigations, case reports, non-randomized controlled trials, and reviews; 2) patients with unclear diagnostic criteria and methods for assessing effectiveness; 3) investigations where the comparison group received CHM treatment; 4) investigations lacking data on reliable endpoint indicators or having inadequately designed experimental protocols; and 5) interventions incorporating additional traditional Chinese medicine physical therapies (e.g., acupuncture, massage, music, etc.).

### 2.3 Literature review and data extraction

Two researchers independently evaluated the gathered literature according to predefined inclusion and exclusion criteria. Information was collected from the selected studies, including the primary author’s name, publication year, sample size, age distribution, gender composition, treatment protocol, treatment duration, form and metabolites of herbal dosage, as well as outcome measures.

### 2.4 Quality assessment

The evaluation of bias risk utilized the bias risk assessment tool suggested in the Cochrane 5.1.0 manual for randomized controlled trials, as outlined by (Cumpston et al., 2019). This assessment included six key aspects: random allocation sequence, concealed allocation scheme, blinding, incomplete outcome data, selective outcome reporting, and “other issues” for methodological quality appraisal. Two researchers performed quality assessment independently, cross-checking each other’s evaluations. Any discrepancies were resolved through consultation with a third researcher.

### 2.5 Data analysis and synthesis

The meta-analysis, conducted using Cochrane Collaboration’s RevMan 5.3 software, involved separate entry of outcome indicators for data processing and analysis. Odds ratio (OR) and standard mean difference (SMD) were utilized to evaluate combined effects for dichotomous outcomes and continuous variables, respectively. Heterogeneity was assessed using the chi-squared test, with I^2^ indicating the degree of heterogeneity. For studies with low heterogeneity (I^2^ < 50%), a fixed-effects model was employed; whereas for those with significant heterogeneity (I^2^ ≥ 50%), subgroup analyses were conducted to explore potential sources. If heterogeneity persisted, a random-effects model was applied for effect size combination, with subgroup analysis based on TCM evidence type, interventions, and intervention time. Publication bias was examined using a funnel plot subsequent to presenting results via a forest plot in the meta-analysis. A significance level of *p* < 0.05 was used to determine statistical significance.

### 2.6 Metabolites of Chinese medicines and their mechanisms of action

The compositions of the formulations and patented drugs are detailed in Supplementary Table S2. The frequency analysis of each CHM is shown in Supplementary Table S3. Network pharmacology analysis was performed on CHMs with a frequency of at least five to identify the main active metabolites and disease targets.

#### 2.6.1 Target identification of Chinese herbal metabolites

Active metabolites from Chinese herbal medicine were gathered using the Traditional Chinese Medicine Systems Pharmacology Database (TCMSP) analysis platform, adhering to criteria of oral bioavailability (OB) > 30% and drug-likeness (DL) ≥ 0.18. Subsequently, the corresponding targets of these active metabolites were assembled and refined utilizing UniProt data, excluding non-human genes and redundant or ineffective targets.

#### 2.6.2 Identification of disease-related targets

Keyword searches for “irritable bowel syndrome,” “depression,” and “anxiety” were performed using the GeneCards database and DisGeNET to retrieve pertinent targets linked with these conditions. Next, all identified targets from these databases were consolidated in Excel, eliminating duplicate genes. The gathered information was then cross-referenced and refined utilizing the UniProt database to ensure accurate gene information for disease targets.

#### 2.6.3 Drug-disease target prediction

The acquired targets of drug metabolites were mapped against disease targets, and then a Venn diagram was generated to obtain the intersecting genes. These intersection targets were inputted into Cytoscape software (version 3.9.0) to construct the herb-metabolite-target network. Additionally, the primary potential mechanisms of action of the top 10 main agents were summarized from the PubMed database.

#### 2.6.4 Constructing a CHM-disease target protein‒protein interaction network

The TCM-disease targets were inputted into the STRING online software, with *Homo sapiens* selected as the species for filtering conditions. This process facilitated the construction of a protein-protein interaction network (PPI network) for drug-disease interactions. A minimum interaction score of 0.4 was set. The degree of each node in the network indicates the protein’s significance in interactions, with a higher number of connections reflecting greater importance within the PPI network.

## 3 Results

### 3.1 Literature search results

**FIGURE 1:**
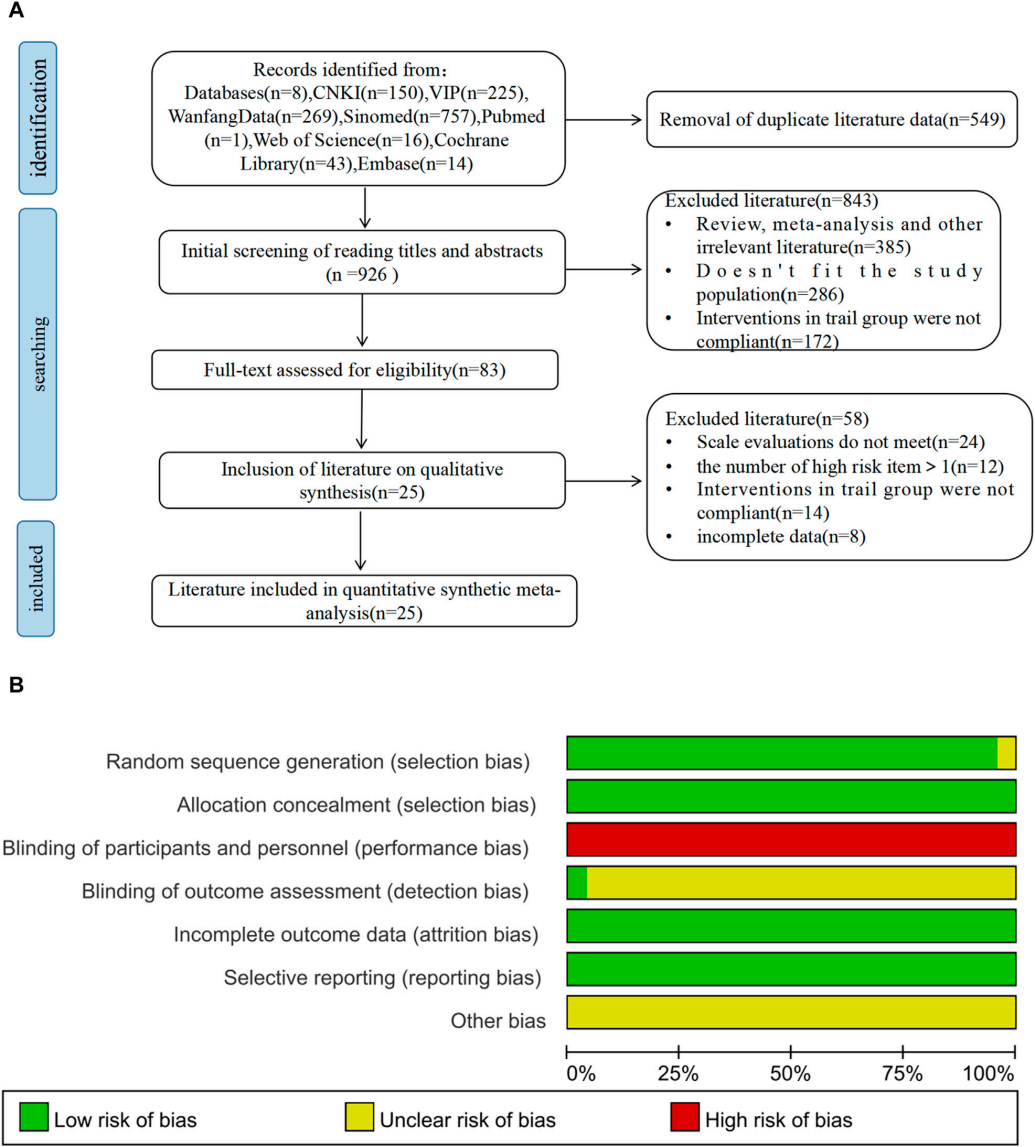
PRISMA flow diagram and risk-of-bias assessment; **(A)** literature screening process; **(B)** risk-of-bias summary.

A total of 1,475 relevant original studies were retrieved from eight databases, including the China National Knowledge Internet (150), VIP (225), Wan Fang (269), SinoMed (757), PubMed (1 study), Web of Science (16), Cochrane Library (43), and Embase (14). A total of 549 duplicates were eliminated. After excluding 843 articles by screening the title and abstract, 83 articles remained for full-text analysis. In total, 58 papers were rejected, and 25 items were included in this study (Figure 1).

### 3.2 Study characteristics and quality

The selected RCTs included 2055 people, 1,028 of whom were in the trial groups and 1,027 of whom were in the control groups. The baseline information for the trial and control groups was similar. The treatment course in all studies varied from 2 weeks to 3 months. The main characteristics of the included studies are summarized in Table 1.

**TABLE 1:** Basic characteristics of included articles.

Study(year)	Diagnostic criteria	N (male/female), mean age (years)		Interventions		Duration of treatment	Outcome index
	IBS-D	Control group	Trial group	Control group	Trial group		
Cai 2020	Rome IV	32(14/18)46.9 ± 15.7	32(17/15)49.5 ± 13.7	Pinaverium Bromide	Western Medicine plus Anchang decoction	8 weeks	①②⑦
Chen 2024	Rome IV	30(11/19) 47.21 ± 7.75	30(14/16) 45.81 ± 7.10	Trimebutine Maleate	Li Pi Qu Shi Fang	4 weeks	①②⑤⑥⑦⑨
Ding 2021	Rome IV	50(27/23) 50.31 ± 9.65	50(26/24) 50.23 ± 9.56	Probiotic drug	Western Medicine plus Hepatogastric Dampening Tablets	4 weeks	③④⑤⑥⑧⑨
Feng 2021	Rome IV	35(17/18) 37.94 ± 1.71	34(15/19) 39.59 ± 1.76	Glutamin Entersoluble Capsules, Trimebutine Maleate	Jian Pi Shen Shi Fang	8 weeks	①②⑥⑦⑧⑨
Fu 2013	RomeⅡ	68(24/44) 39.50 ± 7.29	68(22/46)38.37 ± 4.08	Trimebutine Maleate	Shugan Lipi Zhixie decoction	8 weeks	①②⑥⑦⑧
Gu 2022	Rome IV	30(14/16) 41.0 ± 11.95	30(17/13) 42.77 ± 12.72	Glutamin Entersoluble Capsules	Baishi Wenpi decoction	4 weeks	③④⑥⑦⑧
Guo 2019	Rome IV	34(15/19) 39.02 ± 10.38	33(13/20) 39.34 ± 10.14	Trimebutine Maleate, Probiotics	Western Medicine plus Gu Chang Zhi Xie Wan	2 weeks	①②⑥⑦⑧⑨
He 2021	Rome IV	30(14/16) 40.80 ± 14.50	30(18/12) 45.10 ± 11.80	Pinaverium Bromide	Chaihu Guizhi Gangjiang decoction	4 weeks	①②⑤⑥⑦⑨
Li 2021	Rome IV	30(10/20) 43.77 ± 11.84	30(13/17) 45.30 ± 13.05	Probiotic	Shugan Hezhong decoction	4 weeks	①②⑥⑦⑧
Liu1 2020	Rome IV	33(20/13) 41.94 ± 12.04	34(23/11) 43.23 ± 12.04	Trimebutine Maleate, Flupentixol and melitracen	Tongxie Lizhong decoction	4 weeks	①②⑤⑥⑦⑨
Liu2 2022	Rome IV	47(19/28) 45.38 ± 13.29	47(21/26) 44.62 ± 15.45	Otilonium Bromide, Probiotics	Western Medicine plus Changning Tang Granules	4 weeks	①②⑤⑥⑦⑨
Lu 2021	Rome IV	59(30/29) 73.26 ± 4.15	59(28/31) 73.41 ± 4.28	Montmorillonite powder, Pinaverium Bromide, Domperidone	Western Medicine plus Xiangsha Liujunzi Decoction	4 weeks	③④⑦⑨
Majing 2019	Rome IV	59(33/26) 38.95 ± 12.28	60(32/28) 37.00 ± 10.72	Pinaverium Bromide	Jieyu Tiaochang decoction	2 months	①②⑥⑦⑧⑨
Mou 2021	Rome III	44(18/26) 36.52 ± 2.62	44(20/24) 36.67 ± 2.65	Glutamine Granules	Western Medicine plus Baishi Wenpi decoction	1 month	③④⑥
Nie 2014	Rome III	30/49.80 ± 12.77	30/49.80 ± 12.77	Pinaverium Bromide	Shugan Jianpi Decoction	6 weeks	③④⑥⑦
Su 2022	Rome IV	30(14/16) 45.69 ± 13.81	30(15/15) 45.33 ± 14.39	Probiotic drug	Peitu Shunmu Tang	4 weeks	①②⑤⑥⑦⑨
Sun 2020	Rome IV	30(18/12) 38.93 ± 11.91	30(20/10) 40.87 ± 11.10	Pinaverium Bromide	Jiawei Lichang	4 weeks	③④⑦⑧⑨
Wu 2019	Rome IV	32(13/19) 35.91 ± 8.59	33(13/20) 38.03 ± 9.36	Probiotic drug	Pingwei Capsules	4 weeks	①②⑥⑦⑧⑨
Xu 2017	Rome III	31(18/13) 41.06 ± 12.92	31(20/11) 45.29 ± 10.67	Pinaverium Bromide	Shugan Fupi Huashi Decoction	4 weeks	③④⑤⑥⑦⑨
Yang 2021	Rome IV	30(14/17) 38.70 ± 12.74	30(16/14) 42.77 ± 12.44	Probiotic drug	Jiawei Jiaotai Wan	4 weeks	①②⑤⑨
Zhang1 2016	Rome III	60(26/34) 38.6	60(29/31) 39.2	Pinaverium Bromide	Modified Danggui Shaoyao Powder	4 weeks	③④⑦⑨
Zhang2 2023	Rome IV	59(28/31) 36.04 ± 5.47	59(27/32) 36.69 ± 5.68	Pinaverium Bromide	Western Medicine plus Tongxie Yaofang and Wandai decoction	8 weeks	①②⑥⑦
Zhou 2020	Rome III	30(17/13) 36.23 ± 4.36	30(18/12) 36.89 ± 4.04	Montmorillonite powder, Pinaverium Bromide	Modified Tongxie Yaofang	4 weeks	③④⑥⑦⑧⑨
Zhu1 2013	Rome III	43(16/27) 32.23 ± 11.41	43(19/24) 31.69 ± 10.74	Pinaverium Bromide	Shugan Lipi Fang	3 months	①②⑦
Zhu2 2015	Rome III	71(30/41) 40.72 ± 8.25	71(27/44) 41.24 ± 7.82	Pinaverium Bromide, Loperamide	Western Medicine plus Chaihu Shugan San and Tongxie Yaofang	8 weeks	③④⑦

Note: Indicators of outcomes: ①HAMD②HAMA③SDS④SAS⑤IBS-SSS⑥TCM symptom score⑦Clinical efficacy⑧Recurrence rate⑨Adverse effects.

Among the 25 studies, 15 studies (Fu and Xu, 2013; Zhu, 2013; Guo, 2019; Majing, 2019; Wu, 2019; Cai et al., 2020; Liu, 2020; Feng, 2021; He et al., 2021; Li, 2021; Yang, 2021; Liu, 2022; Su and Zhang, 2022; Zhang, 2023; Chen et al., 2024) used the HAMD and HAMA scales to assess anxiety and depressive conditions, while 10 studies (Nie et al., 2014; Zhu et al., 2015; Zhang, 2016; Xu, 2017; Sun et al., 2020; Zhou and Chu, 2020; Ding et al., 2021; Lu and Wang, 2021; Mou, 2021; Gu and Xu, 2022) employed the SDS and SAS scales for the same purpose. Among the eight studies (Zhu et al., 2015; Guo, 2019; Cai et al., 2020; Ding et al., 2021; Lu and Wang, 2021; Mou, 2021; Liu, 2022; Zhang, 2023), the trial group received a combination of Western medicine and CHM treatment. In contrast, in 17 studies (Fu and Xu, 2013; Zhu, 2013; Nie et al., 2014; Zhang, 2016; Xu, 2017; Majing, 2019; Wu, 2019; Liu, 2020; Sun et al., 2020; Zhou and Chu, 2020; Feng, 2021; He et al., 2021; Li, 2021; Yang, 2021; Gu and Xu, 2022; Su and Zhang, 2022; Chen et al., 2024), the trial group was treated solely with CHM. All control groups received conventional Western medicine treatment.

All studies incorporated in this analysis employed random assignment. Among them, 24 studies utilized either the random number table method or computerized randomization grouping, while one study (Zhou and Chu, 2020) did not provide specific details regarding the process of randomization grouping. Two studies (Li, 2021; Chen et al., 2024) implemented allocation concealment, whereas 23 studies did not clearly state its use. Complete data and reliable results were accessible for all included articles. No disparities were observed in the baseline data of these studies. The outcomes of the risk of bias assessment are depicted in Figure 1.

### 3.3 Meta-analysis of Chinese medicine for the treatment of diarrheal irritable bowel syndrome patients with anxiety and depression

In the majority of studies, significant improvements were observed in HAMA, HAMD, SDS, SAS, IBS-SSS, and TCM symptom scores within the trial group. Consequently, the primary outcome indicators were consolidated to elucidate the efficacy of CHM in treating IBS-D with anxiety and depression.

#### 3.3.1 Clinical effectiveness

**FIGURE 2:**
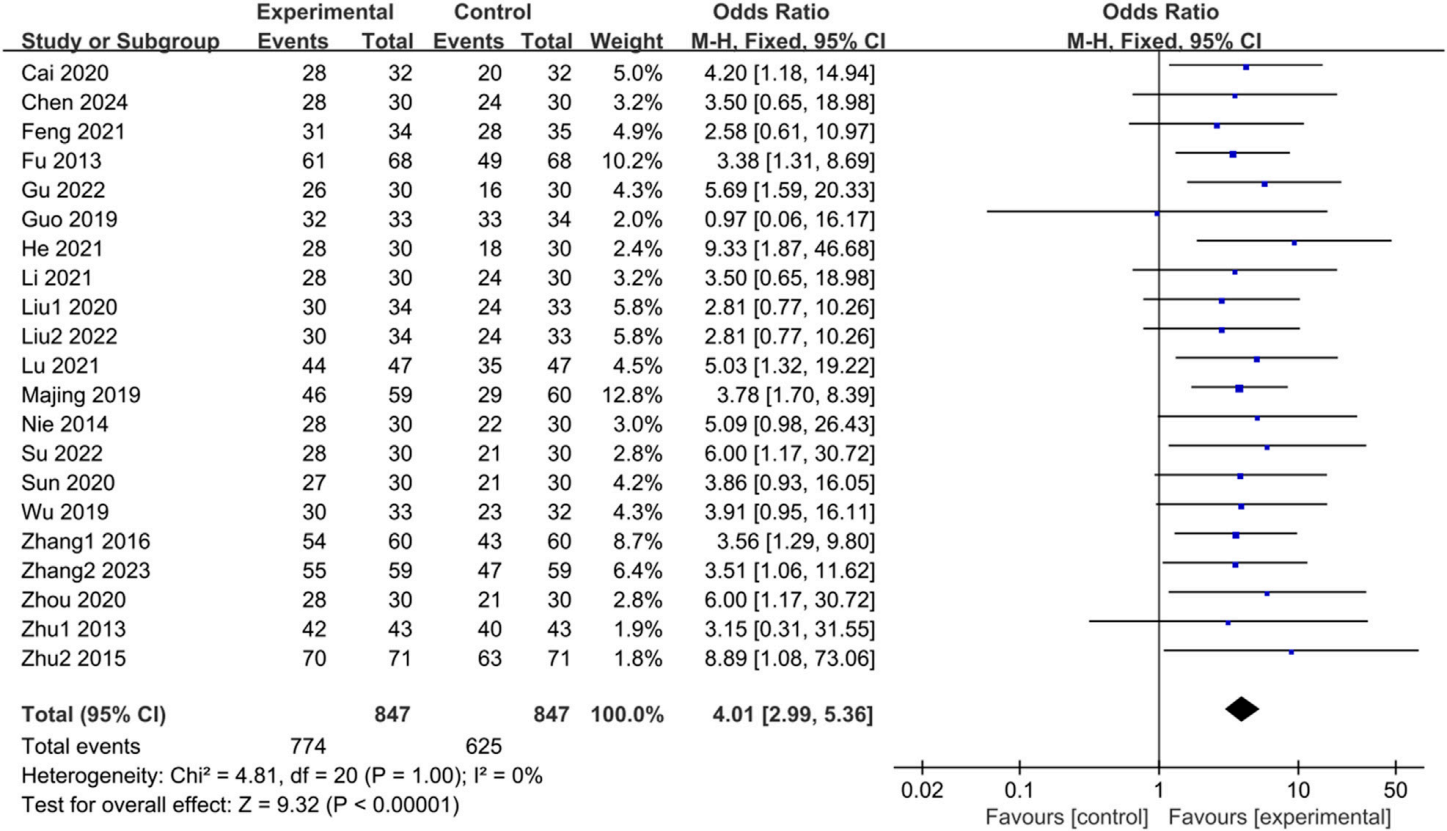
Forest plot comparing the clinical effectiveness of the trial group and the control group after treatment.

There is a pressing need for more standardized efficacy assessment criteria in treating IBS-D. The included studies conducted this investigation to establish their judgment criteria for determining treatment effectiveness. They categorized effective indicators such as cure, apparent effect, and effectiveness, while ineffective indicators were classified accordingly. A total of 21 studies (Fu and Xu, 2013; Zhu, 2013; Nie et al., 2014; Zhu et al., 2015; Zhang, 2016; Guo, 2019; Majing, 2019; Wu, 2019; Cai et al., 2020; Liu, 2020; Sun et al., 2020; Zhou and Chu, 2020; Feng, 2021; He et al., 2021; Li, 2021; Lu and Wang, 2021; Liu, 2022; Su and Zhang, 2022; Gu and Xu, 2022; Zhang et al., 2023; Chen et al., 2024) reported clinical effectiveness rates. The heterogeneity test results (*p* = 1.00, I^2^ = 0%) indicated no statistical heterogeneity among the studies; thus, a fixed-effects model was utilized for the combined analysis. The difference was statistically significant in the test of combined statistics (Z = 9.32, *p* < 0.00001, OR = 4.01, 95% CI [2.99,5.36], I^2^ = 0%), suggesting that the trial group exhibited a higher total effective rate of clinical improvement in IBS-D compared to the control group. These results are illustrated in Figure 2.

#### 3.3.2 HAMD score

**FIGURE 3:**
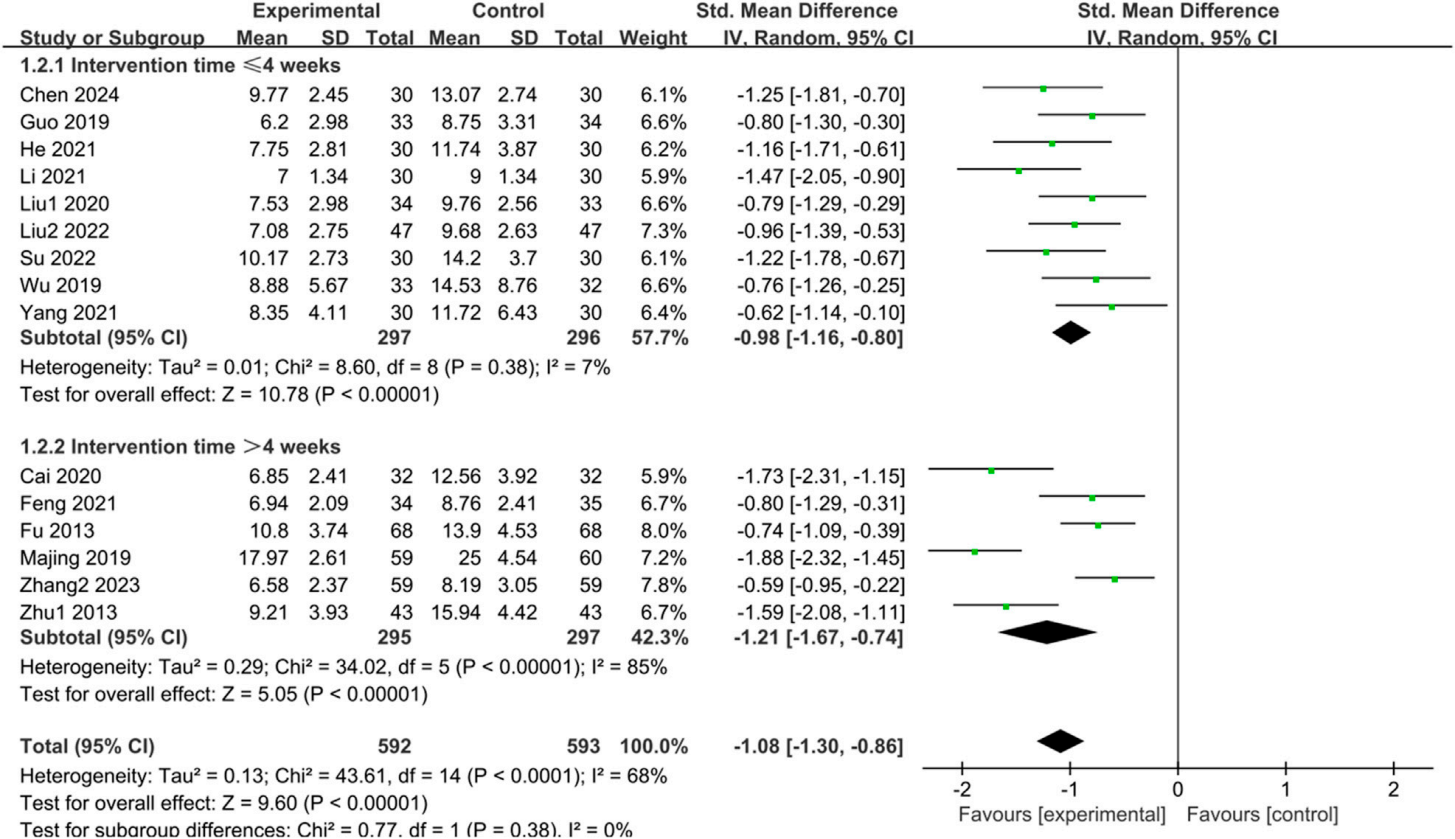
Forest plot comparing the HAMD scores of the trial group and the control group after treatment.

Fifteen studies (Fu and Xu, 2013; Zhu, 2013; Guo, 2019; Majing, 2019; Wu, 2019; Cai et al., 2020; Liu, 2020; Feng, 2021; He et al., 2021; Li, 2021; Yang, 2021; Liu, 2022; Su and Zhang, 2022; Zhang, 2023; Chen et al., 2024) examined HAMD scores of IBS-D patients treated with CHM. Initially, a test for heterogeneity was conducted, revealing significant diversity among the studies (*p* < 0.0001, I^2^ = 68%). Consequently, a random-effects model was applied for the combined analysis. The difference was statistically significant in the combined statistic test (Z = 9.60, *p* < 0.00001). Meta-analysis results indicated that HAMD scores of the trial group were lower than those of the control group (SMD = -1.08, 95% CI [-1.30, −0.86], *p* < 0.00001). Subgroup analyses were carried out to investigate the source of heterogeneity based on intervention time, TCM syndrome of included patients, and interventions (Supplementary Table S4). Subgroup analysis by intervention time revealed: for interventions ≤ 4 weeks, SMD = -0.98, 95% CI [-1.16, −0.80], *p* < 0.00001, I^2^ = 7%; for interventions > 4 weeks, SMD = -1.21, 95% CI [-1.67, −0.74], *p* < 0.00001, I^2^ = 85%. These findings demonstrated significant improvement in reducing HAMD scores with herbal treatment in the trial group compared to the control group, with statistically significant differences. The results are presented in Supplementary Table S4; Figure 3.

#### 3.3.3 HAMA score

**FIGURE 4:**
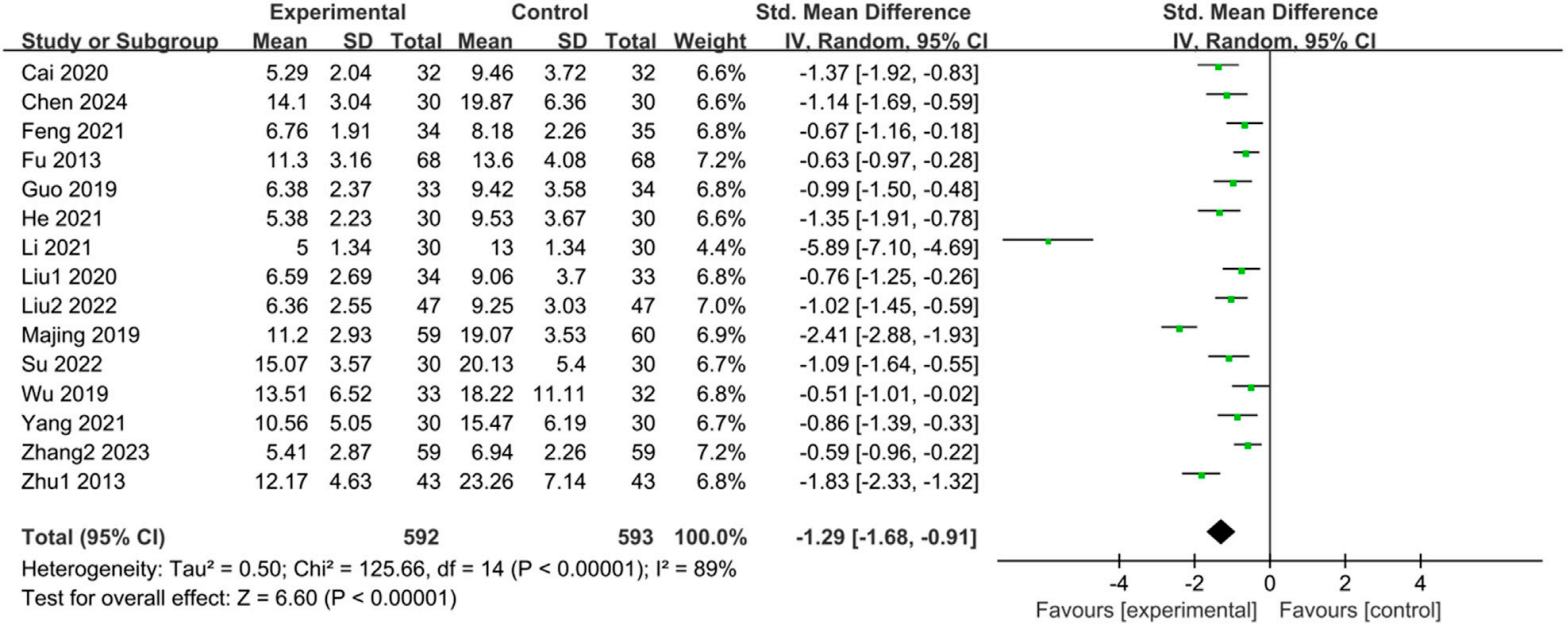
Forest plot comparing HAMA scores of the trial group and the control group after treatment.

Fifteen studies (Fu and Xu, 2013; Zhu, 2013; Guo, 2019; Majing, 2019; Wu, 2019; Cai et al., 2020; Liu, 2020; Feng, 2021; He et al., 2021; Li, 2021; Yang, 2021; Liu, 2022; Su and Zhang, 2022; Zhang, 2023; Chen et al., 2024) reported HAMA scores of IBS-D patients treated with CHM. Initially, a heterogeneity test was conducted, revealing significant heterogeneity among the studies (*p* < 0.00001, I^2^ = 89%). Therefore, a random-effects model was employed for the combined analysis. The difference was statistically significant in the combined statistic test (Z = 6.60, *p* < 0.00001). Subgroup analyses of intervention time, TCM syndrome of included patients, and interventions were conducted to explore the source of heterogeneity, but none of them were found to be significant sources (Supplementary Table S5). A sensitivity analysis was performed to investigate the source of heterogeneity further, and excluding individual studies did not reduce heterogeneity. Meta-analysis results indicated that the HAMA scores of the trial group were lower than those of the control group (SMD = -1.29, 95% CI [-1.68, −0.91], *p* < 0.00001). This suggests that CHM treatment significantly reduced HAMA scores in the trial group compared with the control group, with a statistically significant difference. The results are presented in Supplementary Table S5; Figure 4.

#### 3.3.4 SDS score

**FIGURE 5:**
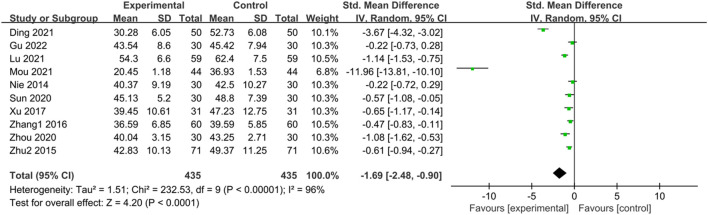
Forest plot comparing the SDS scores of the trial group and the control group after treatment.

Ten studies (Nie et al., 2014; Zhu et al., 2015; Zhang, 2016; Xu, 2017; Sun et al., 2020; Zhou and Chu, 2020; Ding et al., 2021; Lu and Wang, 2021; Mou, 2021; Gu and Xu, 2022) reported SDS scores of IBS-D patients treated with CHM. Initially, a heterogeneity test was conducted, revealing significant heterogeneity among the studies (*p* < 0.0001, I^2^ = 96%). Consequently, a random-effects model was applied for the combined analysis, with a statistically significant difference observed in the combined statistic test (Z = 4.20, *p* < 0.00001). Meta-analysis results showed that SDS scores of the trial group were lower than those of the control group (SMD = -1.69, 95% CI [-2.48, −0.90], *p* < 0.0001). Subgroup analyses were conducted to explore the source of heterogeneity based on intervention time, TCM syndrome of included patients, and interventions (Supplementary Table S6). Subgroup analysis by TCM syndrome revealed: for liver depression and spleen deficiency, SMD = -0.58, 95% CI [-0.76, −0.40], *p* < 0.00001, I^2^ = 12%; for other syndrome types, SMD = -4.01, 95% CI [-6.46, −1.56], *p* < 0.00001, I^2^ = 98%. Subgroup analyses based on interventions indicated: for CHM vs. Western medicine, SMD = -0.51, 95% CI [-0.71, −0.32], *p* < 0.00001, I^2^ = 30%; for CHM + Western medicine vs. Western medicine, SMD = -4.03, 95% CI [-6.17, −1.89], *p* = 0.0002, I^2^ = 98%. The results of the meta-analysis demonstrated that SDS scores of the trial group were lower than those of the control group, indicating that CHM treatment reduced SDS scores in the trial group compared with the control group, with statistically significant differences. The results are presented in Supplementary Table S6; Figure 5.

#### 3.3.5 SAS score

**FIGURE 6:**
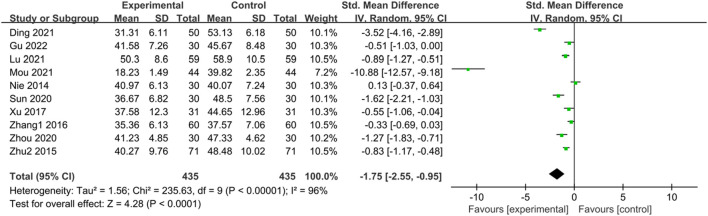
Forest plot comparing SAS scores of the trial group and the control group after treatment.

Ten studies (Nie et al., 2014; Zhu et al., 2015; Zhang, 2016; Xu, 2017; Sun et al., 2020; Zhou and Chu, 2020; Ding et al., 2021; Lu and Wang, 2021; Mou, 2021; Gu and Xu, 2022) reported SAS scores of IBS-D patients treated with CHM. Initially, a heterogeneity test was conducted, revealing significant statistical heterogeneity among the studies (*p* < 0.00001, I^2^ = 96%). Therefore, a random-effects model was applied for the combined analysis, with the combined statistic test yielding a statistically significant difference (Z = 4.28, *p* < 0.0001). Subgroup analyses of intervention time, evidence type of the included patients, and intervention were conducted to explore the source of heterogeneity, but none of them were found to be significant sources (Supplementary Table S7). A sensitivity analysis was performed to investigate the source of heterogeneity further, but no reduction in heterogeneity was observed after individual studies were excluded. Meta-analysis results indicated that the SAS scores of the trial group were lower than those of the control group (SMD = -1.75, 95% CI [-2.55, −0.95], *p* < 0.00001). This suggests that CHM treatment exhibited significant advantages in reducing SAS scores in the trial group compared with the control group, with a statistically significant difference. The results are presented in Supplementary Table S7; Figure 6.

#### 3.3.6 IBS-SSS irritable bowel syndrome severity scale

**FIGURE 7:**
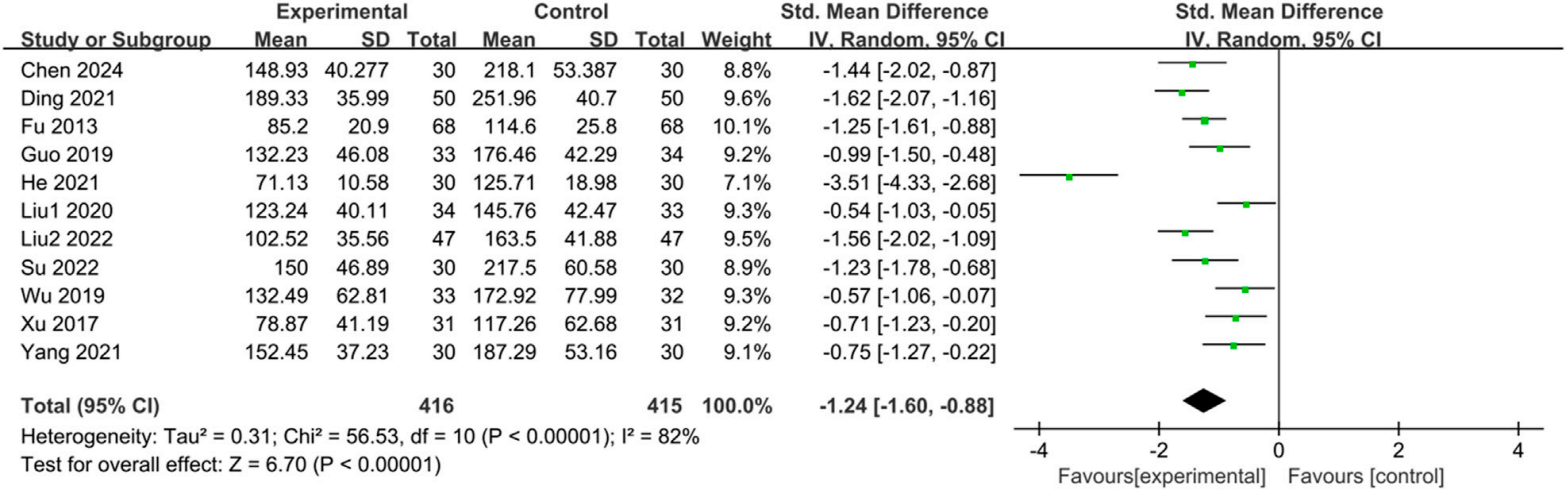
Forest plot comparing the IBS-SSS scores of the trial group and the control group after treatment.

Eleven studies (Fu and Xu, 2013; Xu, 2017; Guo, 2019; Wu, 2019; Liu, 2020; Ding et al., 2021; He et al., 2021; Yang, 2021; Liu, 2022; Su and Zhang, 2022; Chen et al., 2024) reported the IBS-SSS score of IBS-D patients treated with CHM. Initially, a heterogeneity test was conducted, revealing significant statistical heterogeneity among the studies (*p* < 0.00001, I^2^ = 82%). Therefore, a random-effects model was employed for the combined analysis, with the combined statistic test showing a statistically significant difference (Z = 6.70, *p* < 0.00001). Subgroup analyses of intervention time, evidence type of the included patients, and intervention were conducted to explore the source of heterogeneity, but none of them were identified as significant sources (Supplementary Table S8). Sensitivity analysis was performed to investigate the source of heterogeneity further, but heterogeneity could not be reduced after individual studies were excluded one by one. Meta-analysis results indicated that IBS-SSS scores of the trial group were lower than those of the control group (SMD = -1.24, 95% CI [-1.60, −0.88], *p* < 0.00001), suggesting that CHM treatment had a significant advantage in reducing IBS-SSS scores in the trial group compared with the control group, with a statistically significant difference. The results are presented in Supplementary Table S8; Figure 7.

#### 3.3.7 Total clinical symptom scores in Chinese medicine for irritable bowel syndrome (IBS-D)

**FIGURE 8:**
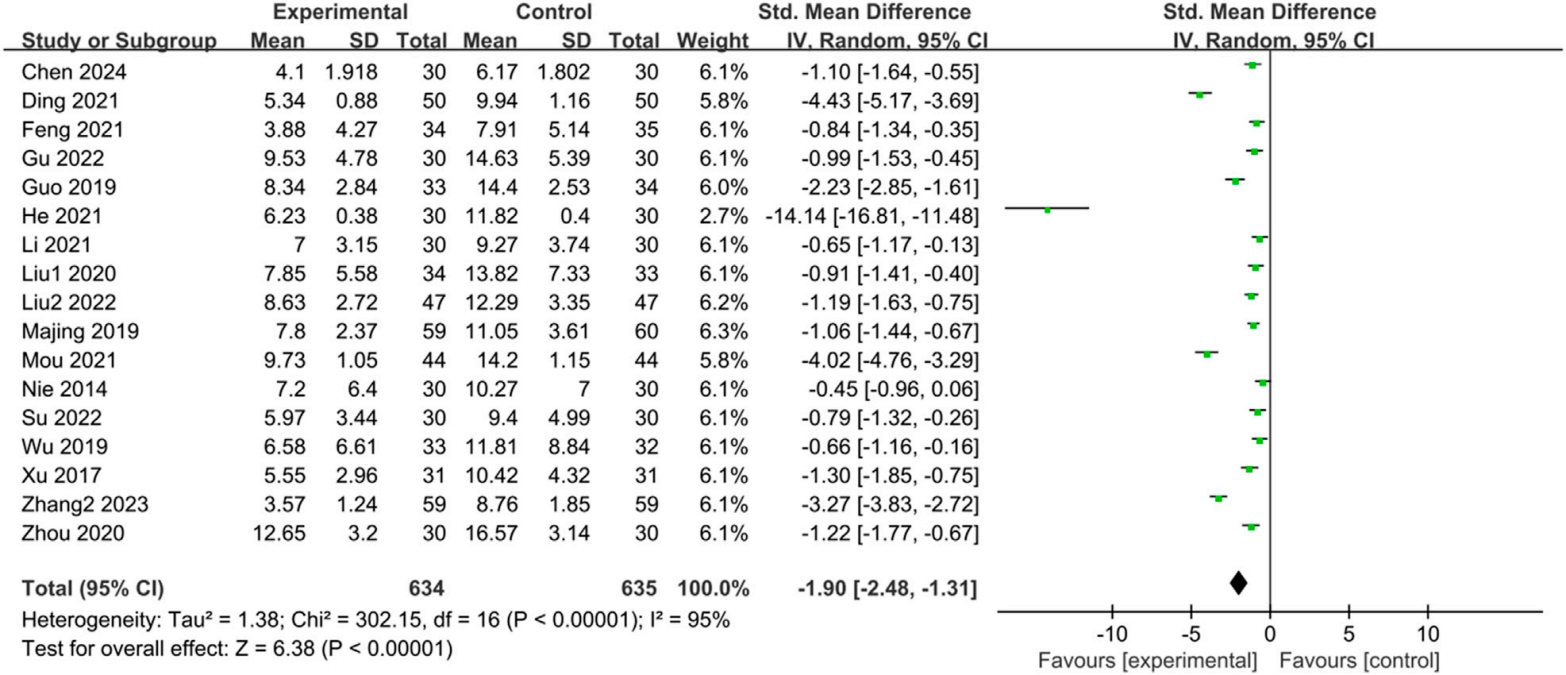
Forest plot comparing TCM symptom scores of the trial group and the control group after treatment.

Seventeen studies (Nie et al., 2014; Xu, 2017; Guo, 2019; Majing, 2019; Wu, 2019; Liu, 2020; Zhou and Chu, 2020; Ding et al., 2021; Feng, 2021; He et al., 2021; Li, 2021; Mou, 2021; Gu and Xu, 2022; Liu, 2022; Su and Zhang, 2022; Zhang, 2023; Chen et al., 2024) reported the TCM symptom scores of IBS-D patients treated with CHM. Due to scoring bias across different studies, significant heterogeneity was observed in the TCM symptom scores. The results of the heterogeneity test (*p* < 0.00001, I^2^ = 95%) indicated statistically significant heterogeneity among the studies. Therefore, a random-effects model was utilized for the merged analysis, with the combined statistic test showing a statistically significant difference (Z = 6.38, *p* < 0.00001). Subgroup analyses of intervention time, evidence type of included patients, and intervention were conducted to explore the source of heterogeneity, but none of them were identified as significant sources (Supplementary Table S9). Sensitivity analysis was employed to further investigate the source of heterogeneity, but heterogeneity could not be reduced after excluding individual studies one by one. Meta-analysis results revealed that TCM symptom scores in the trial group were lower than those in the control group (SMD = -1.90, 95% CI [-2.48, −1.31], *p* < 0.00001), indicating that treatment in the trial group had a significant advantage in reducing TCM symptom scores compared with the control group, with a statistically significant difference. The results are presented in Supplementary Table S9; Figure 8.

#### 3.3.8 Recurrence rate

**FIGURE 9:**
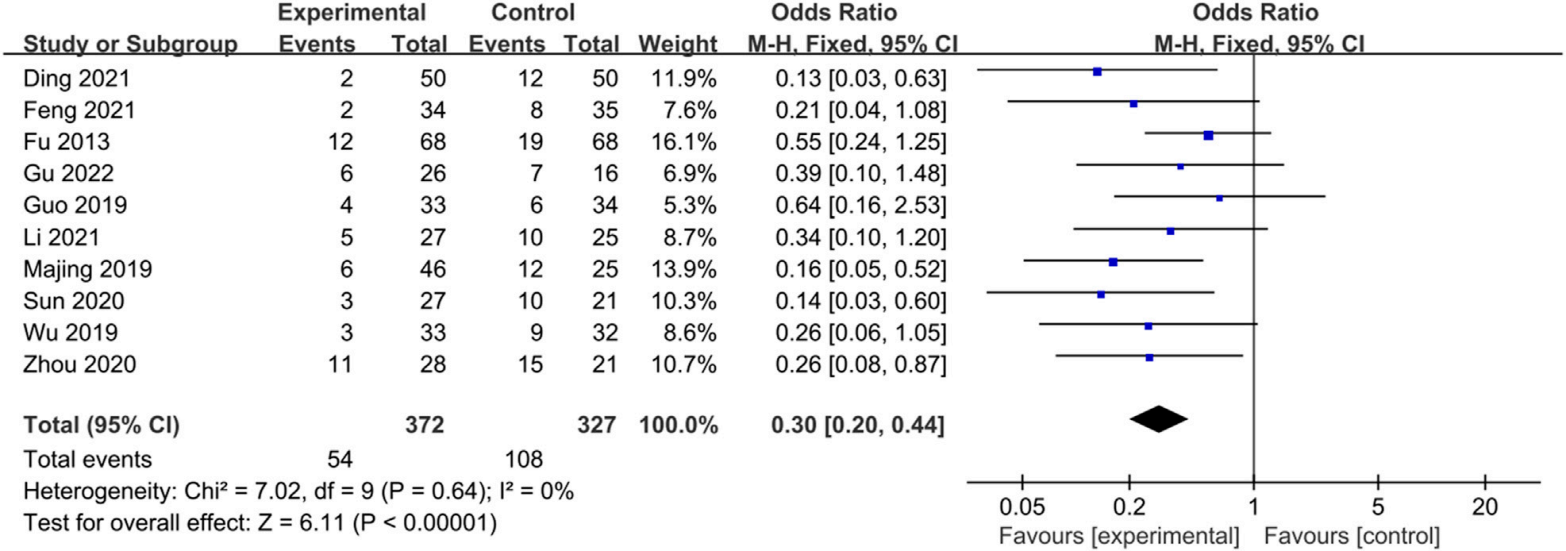
Forest plot comparing the recurrence rates of the trial group and the control group after treatment.

Ten studies (Fu and Xu, 2013; Guo, 2019; Majing, 2019; Wu, 2019; Sun et al., 2020; Zhou and Chu, 2020; Ding et al., 2021; Feng, 2021; Li, 2021; Gu and Xu, 2022) reported recurrence rates, and the heterogeneity test results (*p* = 0.64, I^2^ = 0%), which indicated no statistical heterogeneity among the studies, were combined and analyzed using a fixed-effects model. The results showed that the difference between the trial group and the control group was statistically significant and that CHM treatment could significantly reduce the recurrence rate of IBS-D (Z = 6.11, *p *< 0.00001, OR = 0.30, 95% CI [0.20, 0.44], I² = 0%). The results are shown in Figure 9.

#### 3.3.9 Adverse effects

Fifteen trials (Zhang, 2016; Xu, 2017; Guo, 2019; Majing, 2019; Wu, 2019; Liu, 2020; Sun et al., 2020; Zhou and Chu, 2020; Ding et al., 2021; Feng, 2021; He et al., 2021; Lu and Wang, 2021; Yang, 2021; Su and Zhang, 2022; Chen et al., 2024) assessed adverse occurrences in the 25 included studies. Among them, eleven trials reported no significant adverse reactions. However, four trials (Guo, 2019; Zhou and Chu, 2020; Ding et al., 2021; Lu and Wang, 2021) documented adverse reactions, which included neurological symptoms such as headache, gastrointestinal symptoms such as dry mouth, nausea, vomiting, and constipation, and dermatologic symptoms such as skin rash. The most common adverse reactions are summarized in Table 2.

**TABLE 2:** Occurrence of adverse effects.

Study(year)	Interventions		Adverse effects	
	Control group	Trial group	Control group	Trial group
Guo 2019	Trimebutine Maleate, Probiotics	Western Medicine plus Gu Chang Zhi Xie Wan	No adverse effect	1 case of constipation
Ding 2021	Probiotic drug	Western Medicine plus Hepatogastric Dampening Tablets	2 cases of dry mouth, 1 case of headache, 1 case of rash, and 2 cases of gastrointestinal reaction	2 cases of dry mouth and 2 cases of gastrointestinal reactions
Lu 2021	Montmorillonite powder, Pinaverium Bromide, Domperidone	Western Medicine plus Xiangsha Liujunzi Decoction	1 case of rash, 1 case of nausea, and 1 case of vomiting	3 cases of rash, 2 cases of abdominal pain, 3 cases of constipation, 2 cases of nausea, 2 cases of vomiting, and 1 case of fever
Zhou 2020	Montmorillonite powder, Pinaverium Bromide Tablets	Modified Tongxie Yaofang	3 cases of nausea, vomiting, 2 cases of skin rash, and 3 cases of fever	1 case of nausea, and vomiting

#### 3.3.10 Publication bias

**FIGURE 10:**
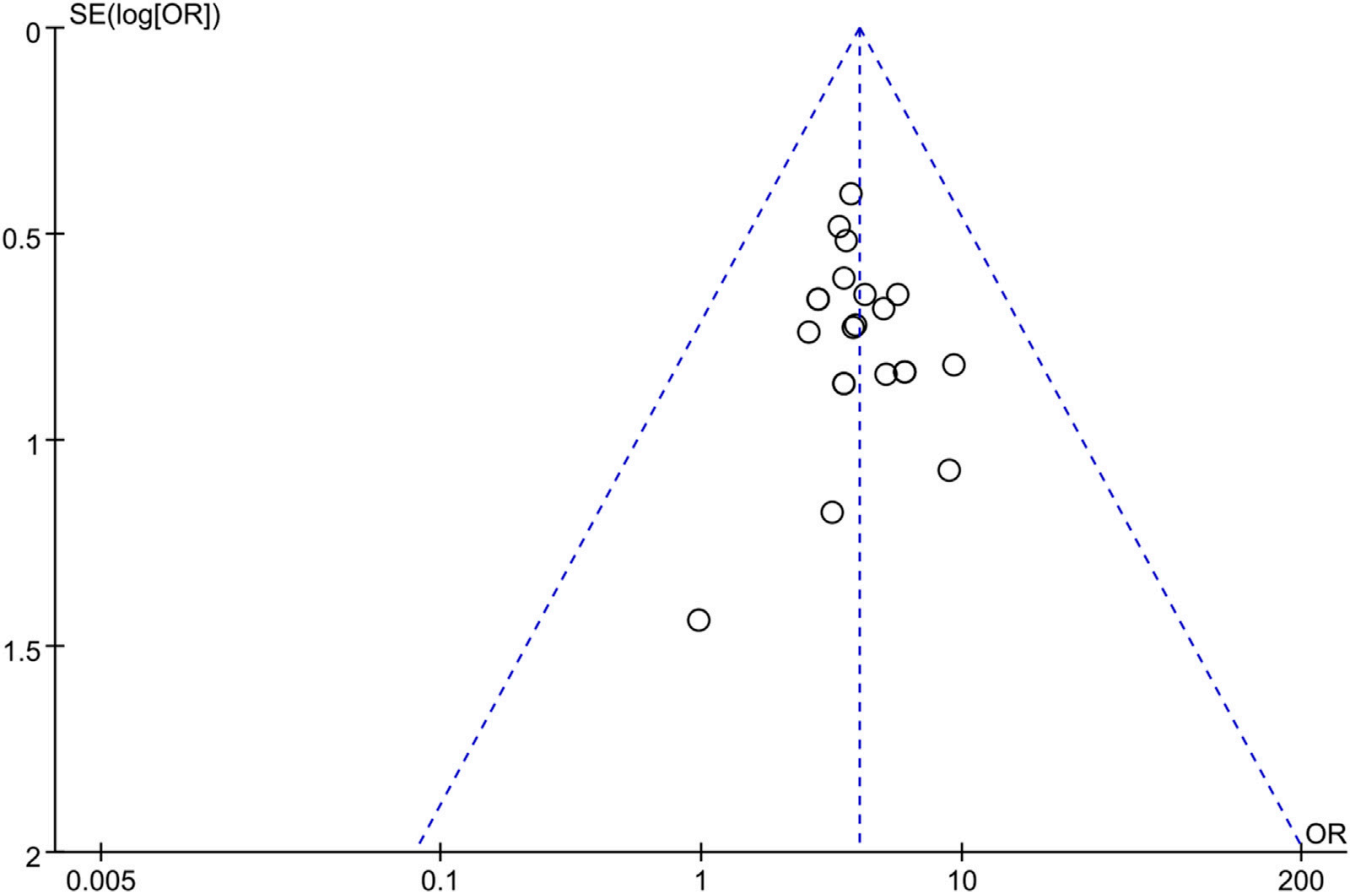
Funnel plot of publication bias in clinical treatment effectiveness rates.

Publication bias was assessed by generating a funnel plot with OR values on the horizontal axis and the standard error (SE) of LogOR on the vertical axis for the primary outcome indicator of this study, which is clinical effectiveness. The funnel plot revealed some asymmetry between the left and right sides, suggesting the presence of publication bias that could influence the combined effect size to some degree. This bias may be attributed to factors such as inconsistent study evaluation, low quality, and small sample size. The results are depicted in Figure 10.

### 3.4 Results of network pharmacology analysis

#### 3.4.1 Active metabolites and treatment targets of CHM

Given the predominance of literature indicating the effectiveness of CHM in treating IBS-D, statistical analysis was conducted to ascertain the frequency of CHM usage and identify commonly employed medications across various groups. CHM metabolites with a frequency of occurrence equal to or greater than five were selected as the primary active metabolites (Supplementary Table S3). The results revealed that *Atractylodes macrocephala Koidz.,* [Asteraceae; Atractylodis macrocephalae rhizoma], *Paeonia lactiflora Pall.*, [Paeoniaceae; Paeoniae radix alba], *Citrus × aurantium L*., [Rutaceae; Citri reticulatae pericarpium], *Glycyrrhiza uralensis Fisch. ex DC*., [Fabaceae; Glycyrrhizae radix et rhizoma praeparata cum melle]*, Bupleurum chinensis DC.,* [Apiaceae; Bupleuri Radix], *Poria cocos* (*Schw.*)*Wolf.,* [Polyporaceae; Poria], *Saposhnikovia divaricata (Turcz. ex Ledeb.) Schischk.,* [Apiaceae; Radix saposhnikoviae], *Dolomiaea costus (Falc.)*., [Asteraceae; aucklandiae radix]*, Codonopsis pilosula (Franch.) Nannf.,* [Campanulaceae; Codonopsis radix], *Dioscorea oppositifolia L.,* [Dioscoreaceae; Dioscoreae rhizoma], *Zingiber officinale Roscoe.,* [Zingiberaceae; Zingiberis rhizoma recens], *Atractylodes lancea (Thunb.) DC*.*,* [Asteraceae; Atractylodis rhizoma], *Prunus mume (Siebold) Siebold & Zucc.,* [Rosaceae; Fructus mume], *Cyperus rotundus L.,* [Cyperaceae; Cyperi rhizoma], *Coptis chinensis Franch.,* [Ranunculaceae; Coptidis rhizoma] are commonly used to treat IBS-D patients with anxiety and depression. After screening, 194 active metabolites of CHM were obtained, along with 294 unique targets. Subsequently, the gene names of the screened targets were converted into gene symbols using the UniProt database.

#### 3.4.2 Disease targets and CHM targets.

Utilizing the GeneCards and DisGeNet disease databases, we conducted a screening for target information associated with IBS-D, anxiety, and depression. The target information from these different diseases was intersected. Subsequently, intersection processing was carried out between the target genes corresponding to the ultimately obtained effective active metabolites of CHM and the disease targets. Consequently, 115 common genes were identified as crucial targets for Traditional Chinese Medicine in treating IBS-D along with anxiety and depression.

#### 3.4.3 Building a “CHM—active metabolites—targets” network

We generated a network diagram of CHM, active metabolites, and targets using Cytoscape 3.9.0. According to topological analysis, active metabolites like beta-sitosterol, stigmasterol, quercetin, kaempferol, luteolin, naringenin, isorhamnetin, nobiletin, wogonin, and formononetin could target factors linked to IBS-D, anxiety, and depression concurrently. Among spleen-tonifying medications, key chemical metabolites include quercetin, kaempferol, luteolin, wogonin, and formononetin. Meanwhile, liver-soothing medications mainly contain beta-sitosterol, stigmasterol, naringenin, isorhamnetin, and nobiletin.

#### 3.4.4 Key target protein interaction network

We utilized a combined_score threshold of 0.4 in the String database to generate a network diagram of target interactions. Following this, the genes underwent network topology analysis using Cytoscape 3.9.0. According to the topological analysis, genes such as IL-6, TNF, PPARG, PTGS2, ESR1, NOS3, MAPK8, and AKT1 are identified as potential targets for CHM in the treatment of IBS-D along with anxiety and depression.

### 3.5 Mechanism of activity

The actions and mechanisms of the top 10 major active metabolites, as presented in Table 3, were investigated by searching the PubMed database.

**TABLE 3:** Actions and mechanisms of the top 10 major active metabolites.

Active metabolite	Source	Models	Related mechanisms	Reference
Beta-sitosterol	*Paeonia lactiflora Pall.*,[Paeoniaceae; Paeoniae radix alba], *Saposhnikovia divaricata (Turcz. ex Ledeb.) Schischk.* [Apiaceae; Radix saposhnikoviae], *Zingiber officinale Roscoe.,*[Zingiberaceae; Zingiberis rhizoma recens], *Prunus mume* (*Siebold*) *Siebold & Zucc.,* [Rosaceae; Fructus mume], *Cyperus rotundus L.,* [Cyperaceae; Cyperi rhizoma], *Citrus × aurantium L.,* [Rutaceae; Aurantii fructus]	IBS: TNBS induced in mice	1.NF-kappa B signaling pathway and MAPK signaling pathway	Lee et al. (2012), Zhai et al. (2023)
		Depression: CUMS mice	1.5-HT, DA and GABA-ergic systems	Yin et al. (2018)
		Anxiety: CRS mice	1.Restraint stress, contextual fear memory, and c-Fos activation in the prefrontal cortex and dentate gyrus	Panayotis et al. (2021)
Stigmasterol	*Bupleurum chinense DC.,*[Apiaceae; Bupleuri radix]*, Codonopsis pilosula (Franch.) Nannf. ,*[Campanulaceae; Codonopsis radix]*, Dolomiaea costus (Falc.) Kasana & A.K.Pandey.,* [Asteraceae; aucklandiae radix]*, Dioscorea oppositifolia L.,* [Dioscoreaceae; Dioscoreae rhizoma]*, Prunus mume (Siebold) Siebold & Zucc.,*[Rosaceae; Fructus mume]*, Cyperus rotundus L.,*[Cyperaceae; Cyperi rhizoma]	Depression: CUMS rat	1.Serotonergic and glutamatergic systems 2.Energy metabolism, HIFα signaling, neurotransmitter, cAMP signaling	Li et al. (2017), Ghosh et al. (2022)
		Anxiety: Elevated plus maze test mice	1.GABAergic mechanism 2.Antagonism of the TRPV1 receptor	Karim et al. (2021), Socała and Wlaź (2016)
Quercetin	*Bupleurum chinensis DC.,*[Apiaceae; Bupleuri Radix], *Glycyrrhiza uralensis Fisch. ex DC*.*,* [Fabaceae; Glycyrrhizae radix et rhizoma praeparata cum melle], *Prunus mume* (*Siebold*) *Siebold & Zucc.,* [Rosaceae; Fructus mume], *Cyperus rotundus L.,* [Cyperaceae; Cyperi rhizoma]	IBS: Male Wistar rats, TNBS solution was infused rat in the distal colon	1.The visceral motor response of PI-IBS animals; and EC cell density and 5-HT level	Qin et al. (2019)
		Depression: CORT-induced/CUMS mice/LPS model	1.Neuroinflammation and oxidative damage	Ma et al. (2021), Adeoluwa et al. (2023), Ge et al. (2023), Xia et al. (2023), Xu et al. (2023)
			2.Glutamatergic system, PI3K-Akt, and BDNF-TrkB signaling pathway	
			3.AHN through the FoxG1/CREB/BDNF signaling pathway; Regulation of BDNF-related imbalanced expression of Copine 6 and TREM1/2	
			4.Modulating monoaminergic transmissions	
			5. Gut microbiota	
		Anxiety:SD rats/zebrafish (*Danio rerio*). MA/LPS model	1.Decreasing ROS, MMP levels, and increasing OCR and ATP production, mitochondrial function and neuroinflammation	Lee et al. (2020), Zhang et al. (2020), Chen et al. (2022), Islam et al. (2022)
			2.Anti-inflammatory effects and appropriate regulation of BDNF and iNOS expression	
			3.GABA Receptor Interaction Pathway	
			4.Neuroinflammation and neuron apoptosis	
Luteolin	*Codonopsis pilosula (Franch.) Nannf.,* [Campanulaceae; Codonopsis radix], *Cyperus rotundus L.,* [Cyperaceae; Cyperi rhizoma]	IBS: WAS rat model	1.Nrf2 signaling pathway, oxidative stress damage in the colon	Xia et al. (2024)
		Depression: CORT model/Sleep Deprivation Stress Model/CUMS rats	1. Inhibition of endoplasmic reticulum stress	Ryu et al. (2022), Wu et al. (2023), Ishisaka et al. (2011)
			2.Glycerophospholipid metabolic pathway in the hippocampus and prefrontal cortex	
			3.Modulating the BDNF/TrkB/ERK/CREB signaling pathway	
		Anxiety: Chronic constriction injury rat model/Sleep Deprivation Stress Model/PTSD model	1.Modulating the BDNF/TrkB/ERK/CREB signaling pathway	Ryu et al. (2022), Sur and Lee (2022), Mokhtari et al. (2023)
			2.Oxidative stress, neurotrophins, and inflammatory factors	
			3.Norepinephrine and serotonin levels	
Kaempferol	*Glycyrrhiza uralensis Fisch. ex DC*.*,*[Fabaceae; Glycyrrhizae radix et rhizoma praeparata cum melle], *Prunus mume* (*Siebold*) *Siebold & Zucc.,* [Rosaceae; Fructus mume], *Cyperus rotundus L.,* [Cyperaceae; Cyperi rhizoma], *Paeonia lactiflora Pall.*,[Paeoniaceae; Paeoniae radix alba], *Bupleurum chinensis DC.,*[Apiaceae; *Bupleuri* Radix]	Depression: CSDS model/CUMS mice/CORT model/PC12 cell	1.Binds to AMPK to promote BDNF production and autophagy enhancement	Gao et al. (2019), Li et al. (2022), Wang et al. (2023), Zhu et al. (2023)
			2.Regulates neuroinflammation, neurotransmitter imbalance, and defective neurogenesis	
			3.Sirt3, activate the mitochondrial antioxidases	
			4.Reduction of oxidative stress, pro-inflammatory cytokines and upregulation of AKT/β-catenin cascade	
		Anxiety: CFC model	1.eCB augmentation via inhibition of the FAAH enzyme	Ahmad et al. (2020)
Naringenin	*Citrus × aurantium L.*[Rutaceae; Citri reticulatae pericarpium], *Glycyrrhiza uralensis Fisch. ex DC*.*,*[Fabaceae; Glycyrrhizae radix et rhizoma praeparata cum melle], *Citrus × aurantium L.,* [Rutaceae; Aurantii fructus]	Depression: Hypoxic stress-induced/CORT mice/CUMS mice/OBX model	1.BDNF and neuroinflammation and neuronal apoptosis	Bansal et al. (2018), Tayyab et al. (2019), Olugbemide et al. (2021), Zhang et al. (2023)
			2.Modulating oxido-inflammatory insults and NF-kB/BDNF expressions	
			3.Acetylcholinesterase activity, oxidative stress and release of pro-inflammatory cytokines	
			4.Restoring alterations in kynurenine pathway via its antioxidant and anti-inflammatory potential	
		Anxiety: male mice/Zebrafish(*Danio rerio*) SDS mice//Iron-Induced	1.Mitochondrial Dysfunctions, Ectonucleotidases and Acetylcholinesterase Alteration Activities	Chtourou et al. (2015), Umukoro et al. (2018), Nachammai et al. (2021)
			2.Neuroprotective potential	
			3.Inhibition of acetylcholinesterase activity, oxidative stress and release of pro-inflammatory cytokines	
Isorhamnetin	*Bupleurum chinensis DC.,*[Apiaceae; Bupleuri Radix], *Glycyrrhiza uralensis Fisch. ex DC*.*,*[Fabaceae; Glycyrrhizae radix et rhizoma praeparata cum melle], *Cyperus rotundus L.,* [Cyperaceae; Cyperi rhizoma]	Depression: LPS-induced//PC12 cells	1.Antioxidant and anti-inflammatory	Gammoh et al. (2023), Xia et al. (2023), Shi et al. (2018)
			2.Activation of PI3K/Akt and ERK pathways	
			3.Synaptic protein expression	
Nobiletin	*Citrus × aurantium L.,*[Rutaceae; Citri reticulatae pericarpium], *Citrus × aurantium L.,* [Rutaceae; Aurantii fructus]	Depression: LPS model and BV2 cells/CUMS mice	1.Promotion of LPS-induced autophagy and attenuated NLRP3 inflammatory vesicle activation involved in the AMPK pathway	Li et al. (2013), Wang et al. (2020)
			2.Serum corticosterone levels, BDNF, TrkB, and synapsin BDNF-TrkB pathway	
		Anxiety: CMS mice	1.Altered gut microbiome	Tsai et al. (2023)
Wogonin	*Saposhnikovia divaricata (Turcz. ex Ledeb.) Schischk.,* [Apiaceae; Radix saposhnikoviae]	Depression: CORT mice/CUMS mice	1.Restoring the DEPs involved in signal transduction and regulation	Zhang et al. (2019), Lee et al. (2017), Su et al. (2014)
			2.inhibitory MAO-A/MAO-B	
			3.5-HT, DA systems and hippocampal neurogenesis	
		Anxiety: male mice	1.GABA, Central Nervous System Activity, sedation	Hui et al. (2002), Fong et al. (2017)
Formononetin	*Glycyrrhiza uralensis Fisch. ex DC*.*,*[Fabaceae; Glycyrrhizae radix et rhizoma praeparata cum melle]	Depression: CORT mice	1. Reduce neuronal damage and promote neurogenesis, increased GR and BDNF in the hippocampus	Zhang et al. (2022)

Note: Examples of CHM, sources are derived from the results of network pharmacology analysis. 5-HT: 5-hydroxytryptamine. Akt: Protein kinase B. AHN: adult hippocampal neurogenesis. AMPK: adenosine monophosphate-activated protein kinase. ATP: adenosine triphosphate. BDNF: brain-derived neurotrophic factor. cAMP: Cyclic adenosine monophosphate. CFC: Contextual fear conditioning. CMS: Chronic mild stress. Copine 6: Anti-CPNE6, rabbit polyclonal antibody. CORT: Corticosterone. CREB: cAMP, response element binding protein. CRS: Chronic restraint stress. CUMS: Chronic unpredictable mild stress. CORT: Corticosterone. CSDS: chronic social defeat stress. DA: dopamine. DEPs: differentially expressed proteins. eCB: endocannabinoid. ERK: extracellular signal-regulated kinase. FAAH: Fatty acid amide hydrolase. FoxG1: Forkhead box G1. FST: Forced swimming test. GABA: Gamma-aminobutyric acid. GR: glucocorticoid receptor. iNOS: inducible nitric oxide synthase. IL-1β; Interleukin-1, beta. LPS: lipopolysaccharide. MA: Methamphetamine-induced. MAO-A: Monoamine oxidase-A. MAO-B: Monoamine oxidase-B. MMP: mitochondrial membrane potential. NF-κB: nuclear factor kappa-B. NLRP3: NOD-like receptor thermal protein domain associated protein 3. NR3C1: Nuclear receptor subfamily three group C member 1. Nrf2: nuclear factor erythroid-2-related factor 2. OBX: olfactory bulbectomy. OCR: oxygen consumption rate. PFC: prefrontal cortex. PI3K: Phosphatidylinositol-4, 5-bisphosphate 3-kinase. PKA: protein kinase A. PSD95: postsynaptic density protein-95. PTSD: Posttraumatic stress disorder. ROS: reactive oxygen species. SDS: Social defeat stress. Sirt3: Recombinant Sirtuin 3. TGF-β: transforming growth factor-β. TNBS: trinitro-benzenesulfonic acid. TNF-α: tumor necrosis factor-α. TREM1/2: Triggering receptor expressed on myeloid cells 1/2. TrkB: tyrosine kinase receptor B. TRPV1: Transient receptor potential vanilloid 1. WAS: water avoidance stress.

#### 3.5.1 Study of potential mechanisms

The PubMed database was queried to elucidate the mechanism of action of the primary active metabolites (top 10). As indicated in Table 3, beta-sitosterol, quercetin, and luteolin were investigated in studies related to IBS-D, anxiety, and depression. Additionally, studies focusing on anxiety and depression have explored stigmasterol, naringenin, nobiletin, wogonin, and kaempferol. Commonly utilized animal models for IBS-D include the maternally separated (MS) IBS-D rat model, the 2,4,6-trinitrobenzene sulfonic acid (TNBS)-induced post-inflammatory IBS-D rat model, and models induced by water avoidance stress (WAS). Furthermore, animal models commonly used for depression and anxiety include multiple-stress mice, LPS/CORT mice, and *Danio rerio* models.

Table 3 illustrates the mechanisms attributed to these ten active metabolites concerning IBS-D, anxiety, and depression. These metabolites effectively address visceral sensitivity, normalize gastrointestinal dynamics, diminish inflammatory responses, and regulate mood. They exhibit anti-inflammatory and antioxidative stress effects while maintaining neurotransmitter balance. IBS-D is currently associated with various mechanisms like gut microbiota, visceral hypersensitivity, low-grade inflammation, and brain-gut axis interactions. Beta-sitosterol, quercetin, and luteolin alleviate intestinal symptoms by targeting visceral hypersensitivity, gastrointestinal infections, inflammation, and psychosocial aspects. They regulate sensory nerve pathways and neurotransmitter signaling, reducing gastrointestinal discomfort such as abdominal pain. Additionally, their anti-inflammatory properties alleviate chronic gastrointestinal inflammation, easing symptoms like diarrhea and rectal bleeding. Moreover, these metabolites modulate neuroendocrine and neuroimmune pathways linked to stress response, anxiety, and depression, thereby improving psychosocial wellbeing and gastrointestinal health overall.

With the exception of stigmasterol, wogonin, and formononetin, the other active metabolites exhibit potent anti-inflammatory and antioxidant properties. These metabolites inhibit the release of inflammatory mediators, diminish inflammatory cell activity, and improve immune system function. They play a crucial role in mitigating oxidative stress, a contributing factor to various diseases. Kaempferol, abundant in plant-based foods, demonstrates robust anti-inflammatory effects by inhibiting pro-inflammatory cytokines and scavenging free radicals, thereby safeguarding against tissue damage induced by oxidative stress. Naringenin scavenges free radicals and reactive oxygen species, shielding cells from oxidative harm and modulating inflammatory signaling pathways. Isorhamnetin suppresses NF-κB activation and dampens inflammatory gene expression while possessing antioxidant properties that protect against cellular damage induced by oxidative stress, particularly in gastrointestinal and neuronal tissues affected by psychological stressors. Nobiletin, present in citrus CHM, alleviates gastrointestinal inflammation, restores intestinal barrier integrity, and mitigates stress-induced neuroinflammation and mood disorders by modulating inflammatory pathways and oxidative stress responses.

The imbalance of adrenocorticotropic hormone, serotonin, dopamine, and tryptophan contributes to neurochemical disturbances seen in anxiety and depression. Beta-sitosterol, stigmasterol, and quercetin play a role in modulating these neurotransmitter systems, showing promise in managing anxiety and depression. Isorhamnetin influences synaptic protein expression, impacting synaptic communication. Stigmasterol, quercetin, luteolin, kaempferol, naringenin, nobiletin, and formononetin regulate neurogenesis and brain-derived neurotrophic factor (BDNF) expression, supporting neuronal growth, synaptic plasticity, and mood stability. Quercetin and naringenin alleviate depression and anxiety by addressing mitochondrial dysfunction, restoring mitochondrial function, and reducing oxidative stress. The therapeutic mechanisms of CHM metabolites in IBS-D with concurrent anxiety and depression involve complex signaling pathways, including NLRP3 inflammatory vesicle, cAMP/PKA, BDNF-TrkB, PI3K/AKT/NF-κB, MEK/ERK, and FoxG1/CREB/BDNF pathways. Understanding these intricate signaling networks could inform the development of new therapeutic strategies for managing gastrointestinal disorders and associated neuropsychiatric symptoms.

## 4 Discussion

There is increasing evidence indicating a high prevalence of depression and anxiety among patients with IBS-D. Current medical approaches for treating IBS-D include pharmacological symptomatic treatments, dietary adjustments, and psychotherapies. Chinese medicine has emerged as a significant modality in managing IBS-D with comorbid anxiety and depression, offering enhanced efficacy and fewer side effects compared to conventional treatments. Several clinical studies have highlighted its precise efficacy and safety. However, further validation of CHM’s effectiveness in treating IBS-D with anxiety and depression is needed, particularly due to concerns regarding methodological quality.

This study conducted a comprehensive review of domestic and international research on treating IBS-D with depression and anxiety using CHM. The analysis included 25 studies, comprising 22 CHM metabolite prescriptions and involving 2055 patients. The findings revealed that the CHM-based trial group exhibited superior efficacy compared to the control group (*p* < 0.05). Additionally, the trial group showed significant improvements in depression scale scores (HAMD and SDS) and anxiety scale scores (HAMA and SAS) compared to the control group (*p* < 0.05), indicating the potential of CHM treatment to ameliorate mood disorders in IBS-D patients. Moreover, CHM treatment demonstrated advantages in alleviating clinical gastrointestinal discomfort, accompanying symptoms (evaluated by IBS-SSS and total TCM symptom scores), and improving patients’ quality of life (*p* < 0.05). The recurrence rates in the trial group were lower (14.52%) compared to the control group (33.03%), suggesting a reduced recurrence risk of irritable bowel syndrome with depression and anxiety following CHM intervention. In conclusion, CHM exhibits clinical effectiveness in managing IBS-D with depression and anxiety without increasing the risk of adverse effects.

The gastrointestinal tract operates under a complex network involving central, autonomic, and enteric nervous systems, making it susceptible to influences from adverse emotions and psychological factors (Aburto and Cryan, 2024). This disruption in the brain-gut axis can lead to gastrointestinal dysfunction due to imbalances between the hypothalamus and limbic system, as well as reduced vagal nerve excitability. The mechanism of TCM in the management of IBS-D involves various aspects. Firstly, Chinese herbs alleviate patients’ symptoms by regulating intestinal function, modulating intestinal motility and peristalsis, possibly affecting the smooth muscle of the intestines to promote the normalization of peristalsis. Secondly, TCM can rebalance the yin and yang imbalance in the body by regulating the neuroendocrine system. Through the microbiota-intestinal-brain axis, CHM can ameliorate patients’ abdominal discomfort and psychological symptoms. Additionally, certain CHM enhance the function of the digestive system, promoting the absorption of nutrients, thereby alleviating symptoms in patients with IBS-D. Finally, several herbal medicines possess anti-inflammatory and antioxidant properties, aiding in reducing inflammation reactions in the intestinal mucosa, improving the intestinal environment, and thereby alleviating symptoms. CHM offers a promising approach to managing IBS-D alongside depression and anxiety by employing a multifaceted, multitarget strategy. To investigate the utilization of CHM further, this study analyzed commonly used herbal medications using network pharmacology. The findings revealed several frequently used herbs, including *A. macrocephala Koidz.,* [Asteraceae; Atractylodis macrocephalae rhizoma], *P. lactiflora Pall.,* [Paeoniaceae; Paeoniae radix alba], *G. uralensis Fisch. ex DC.,* [Fabaceae; Glycyrrhizae radix et rhizoma praeparata cum melle], *Citrus × aurantium L.,* [Rutaceae; Citri reticulatae pericarpium], *P. cocos* (*Schw.*)*Wolf.,* [Polyporaceae; Poria], *Bupleurum chinense DC.,* [Apiaceae; Bupleuri radix], *D. oppositifolia L.,* [Dioscoreaceae; Dioscoreae rhizoma], *C. pilosula (Franch.) Nannf.,* [Campanulaceae; Codonopsis radix], *D. costus (Falc.) Kasana & A.K.Pandey.,* [Asteraceae; aucklandiae radix],*A. lancea (Thunb.) DC.,* [Asteraceae; Atractylodis rhizoma], *C. rotundus L.,* [Cyperaceae; Cyperi rhizoma]. These herbs contain active metabolites that, according to network pharmacology, can concurrently target disease-related pathways associated with IBS-D, anxiety, and depression. Notable among these active metabolites are beta-sitosterol, stigmasterol, quercetin, naringenin, luteolin, kaempferol, nobiletin, wogonin, formononetin, and isorhamnetin. These metabolites exhibit potential therapeutic effects on the interconnected targets of IBS-D, anxiety, and depression, underscoring the holistic approach of CHM in addressing these complex conditions.

Herbal metabolites like beta-sitosterol, quercetin, and luteolin are pivotal in addressing the intricate relationship between IBS-D and concurrent depression and anxiety. On the other hand, stigmasterol, naringenin, kaempferol, nobiletin, and wogonin target depression and anxiety symptoms. Additionally, formononetin and isorhamnetin play essential roles in treating depression. Beta-sitosterol, a phytosterol, exhibits anti-inflammatory and immunomodulatory effects. Quercetin, known for its anti-inflammatory, antioxidant, and anticancer properties, positively influences immune function by activating AMP-activated protein kinase (AMPK) (Chiang et al., 2023). Luteolin regulates the Nrf2 signaling pathway, protecting against excessive intestinal motility and diarrhea (Xia et al., 2024). Naringenin enhances cell survival by reducing apoptosis rates induced by CORT (Zhang et al., 2023). Stigmasterol demonstrates anti-inflammatory, antioxidant, and neuroprotective characteristics, potentially alleviating depression and anxiety symptoms by maintaining neurotransmitter balance. Kaempferol exhibits anti-ulcerative colitis effects, suggesting promising therapeutic mechanisms (Qu et al., 2021). Nobiletin inhibits pro-inflammatory cytokines and enzymes like COX-2 and iNOS, scavenges free radicals, and reduces oxidative stress. Wogonin suppresses NF-κB activation and the production of inflammatory mediators such as TNF-α and IL-6. Formononetin displays antioxidant effects by mitigating neuronal damage and promoting neurogenesis (Zhang et al., 2022). Isorhamnetin possesses antioxidant, anti-inflammatory, and neuroprotective attributes, hindering the production of inflammatory cytokines and mediators, reducing oxidative stress, and enhancing neuronal survival and synaptic plasticity. Inflammation significantly contributes to the pathophysiology of IBS-D and its associated psychiatric comorbidities, exacerbating symptoms and fostering mood disorders like depression and anxiety. The bidirectional communication of the gut-brain axis is pivotal, wherein gut-derived inflammatory signals influence central nervous system function and mood regulation. CHM metabolites exhibit anti-inflammatory and neurological protection effects by modulating signaling molecules and oxidative stress, alongside antioxidant properties by neutralizing free radicals and curtailing cellular damage in order to improve diarrhea, abdominal discomfort, and mood in patients.

Based on the intersection of metabolite-disease targets, these CHM can target multiple receptors, including PPARG, PTGS2, ESR1, NOS3, MAPK8,1L-6, TNF, and AKT1, to elicit synergistic treatment of diseases effects. PPARG, expressed in various tissues, regulates lipid catabolism and exhibits anti-inflammatory effects when activated, potentially ameliorating colitis symptoms. PTGS2, encoding a crucial cellular protein, modulates anti-inflammatory responses and immune regulation; thus, impacting mood regulation. AKT1, a ubiquitous intracellular kinase, regulates cell metabolism, survival, and proliferation. Phosphorylated AKT1 can activate NLRP3 inflammatory vesicles, contributing to inflammation in colitis-related diseases (Guo et al., 2014). TNF enhances chemokine and cytokine production, amplifying the inflammatory cascade and organ damage, while IL-6, a key cytokine in inflammation induction and maintenance, may contribute to systemic inflammatory responses and mood dysregulation (Ridker, 2016). ESR1, functioning as a transcription factor, regulates gene expression, affecting processes like cell proliferation, differentiation, and apoptosis. NO, a signaling molecule involved in neurotransmission and immune response, plays a crucial role in inflammation, with NOS3-derived NO potentially impacting mood regulation through its involvement in inflammatory processes. MAPK8 responds to various extracellular stimuli, including stress and cytokines, regulating gene expression implicated in inflammation and neuronal plasticity, thereby modulating depression and anxiety.

In summary, the effectiveness of CHM in treating IBS-D patients with anxiety and depression is evident. Importantly, CHM appears to exert multi-metabolite and multi-targeted effects on signaling pathways involved in various aspects of the biology of IBS-D patients with anxiety and depression, including anti-injury/apoptosis, anti-inflammation, antioxidative stress, and neurotransmitter homeostasis maintenance. CHM may ameliorate symptoms of IBS-D that cooccur with anxiety and depression by addressing different facets of the condition.

## 5 Limitations

Firstly, the quality of the included studies was subpar, characterized by low methodological quality. Most studies lacked details on allocation concealment and blinding, and some exhibited selective reporting bias. In future clinical trials, we aim to adhere to the international CONSORT standards to ensure robust study design and reporting. Secondly, many of the included studies had small sample sizes, diminishing the statistical power of our analysis. Moreover, the absence of rigorous sample size estimation in these studies undermines the validity of the findings. Additionally, variations in conventional interventions introduced clinical heterogeneity, such as differing choices of conventional Western medicine, varying intervention durations, and diverse criteria for evaluating efficacy. Subgroup analyses to identify factors influencing heterogeneity were inadequate. Future research endeavors should prioritize rigorous, multicenter, large-sample size randomized controlled trials to furnish high-quality evidence for clinical practice.

## 6 Conclusion

CHM demonstrates efficacy in ameliorating symptoms associated with irritable bowel syndrome (IBS-D) in individuals suffering from anxiety and depression. The principal mechanisms underlying the actions of these herbal active metabolites likely involve anti-inflammatory and antioxidative stress effects, along with the regulation of neurotransmitter homeostasis and modulation of autophagy.

## Data Availability

The original contributions presented in the study are included in the article/Supplementary Material, further inquiries can be directed to the corresponding author.
