# Supplementary material for: Exploration of the mechanism of Traditional Chinese Medicine for anxiety and depression in patients with diarrheal irritable bowel syndrome based on network pharmacology and meta-analysis
Source: Front Pharmacol. 2024 May 21;15:1404738. doi: 10.3389/fphar.2024.1404738 (PMC11148253; doi:10.3389/fphar.2024.1404738)
Supplement: Supplementary file 1 [file Table1.DOCX]

Supplementary Material

**Exploration of the Mechanism of Traditional Chinese Medicine for Anxiety and Depression in Patients with Diarrheal Irritable Bowel Syndrome Based on Network Pharmacology and Meta-analysis**

Chen Bai^#1^, Junyi Wang^1^,Yifan Wang^1^, Haoqi Liu^1^, Jiaxiu Li^1^,Siyi Wang^1^, Zhen Bai^2^, Rongjuan Guo^3*^,

^1^Dongfang Hospital, Beijing University of Chinese Medicine, Beijing, China

^2^Department of Medical Equipment, The First Affiliated Hospital of Zhengzhou University, Zhengzhou, China

^3^Psychosomatic Department, Dongfang Hospital, Beijing University of Chinese Medicine, Beijing, China

**Supplementary Table S1**. The search strategy

**Pubmed**

| **#** | **Search terms** | **Results** |
| --- | --- | --- |
| 1 | (("Depression"[Mesh]) OR ("Depressive Disorder"[Mesh])) OR (((((((((((((((((Depressive Symptoms[Title/Abstract]) OR (Depressive Symptom[Title/Abstract])) OR (Symptom, Depressive[Title/Abstract])) OR (Emotional Depression[Title/Abstract])) OR (Depressive Disorders[Title/Abstract])) OR (Neurosis, Depressive[Title/Abstract])) OR (Depressive Neuroses[Title/Abstract])) OR (Depressive Neurosis[Title/Abstract])) OR (Depression, Endogenous[Title/Abstract])) OR (Endogenous Depressions[Title/Abstract])) OR (Depression, Neurotic[Title/Abstract])) OR (Depressions, Neurotic[Title/Abstract])) OR (Neurotic Depression[Title/Abstract])) OR (Melancholia[Title/Abstract])) OR (Melancholias[Title/Abstract])) OR (Unipolar Depression[Title/Abstract])) OR (Depression, Unipolarr[Title/Abstract])) OR (Angst[Title/Abstract])) | 296688 |
| 2 | (("Anxiety"[Mesh])) OR ("Anxiety Disorders"[Mesh])) OR (((((((((((((((Social Anxiety[Title/Abstract])) OR (Anxieties, Social[Title/Abstract])) OR (Anxiety, Social[Title/Abstract])) OR (Social Anxieties[Title/Abstract])) OR (Hypervigilance[Title/Abstract])) OR (Nervousness[Title/Abstract])) OR (Anxiousness[Title/Abstract])) OR (Anxiety Disorder[Title/Abstract])) OR (Neuroses, Anxiety[Title/Abstract])) OR (Anxiety Neuroses[Title/Abstract])) OR (Anxiety States, Neurotic[Title/Abstract])) OR (Anxiety State, Neurotic[Title/Abstract])) OR (Neurotic Anxiety State[Title/Abstract])) OR (Neurotic Anxiety States[Title/Abstract])) OR (State, Neurotic Anxiety[Title/Abstract])) | 208358 |
| 3 | #1 AND #2 | 140889 |
| 4 | ("Irritable Bowel Syndrome"[Mesh]) OR (((((((Irritable Bowel Syndromes[Title/Abstract]) OR (Syndrome, Irritable Bowel[Title/Abstract])) OR (Colon, Irritable[Title/Abstract])) OR (Irritable Colon[Title/Abstract])) OR (Mucous Colitides[Title/Abstract])) OR (Mucous Colitis[Title/Abstract])) OR (IBS[Title/Abstract])) OR (IBS[Title/Abstract])) OR (IBS-D[Title/Abstract])) | 15520 |
| 5 | (("Herbal Medicine"[Mesh]) OR ("Medicine, Chinese Traditional"[Mesh])) OR ((((((((((Traditional Chinese Medicine[Title/Abstract]) OR (Zhong Yi Xue[Title/Abstract])) OR (Chinese Traditional Medicine[Title/Abstract])) OR (Chinese Medicine[Title/Abstract])) OR (Traditional Tongue Diagnosis[Title/Abstract])) OR (Tongue Diagnoses, Traditional[Title/Abstract])) OR (Traditional Tongue Diagnoses[Title/Abstract])) OR (Traditional Tongue Assessment[Title/Abstract])) OR (Traditional Tongue Assessments[Title/Abstract])) OR (Chinese herbal medicine[Title/Abstract])) | 55296 |
| 6 | (((randomized[Title/Abstract]) OR (RCT[Title/Abstract])) OR (Clinical Control[Title/Abstract])) OR ("Randomized Controlled Trial" [Publication Type]) | 987058 |
| 7 | #3 AND #4 AND #5 AND #6 | **1** |

**Web of Science**

| **#** | **Search terms** | **Results** |
| --- | --- | --- |
| 1 | (((((((((((((((((((TS=(Depression)) OR TS=(Depressive Disorder)) OR TS=(Depressive Symptoms)) OR TS=(Depressive Symptom)) OR TS=(Emotional Depression)) OR TS=(Depressive Disorders)) OR TS=(Depressive Neuroses)) OR TS=(Depressive Neurosis)) OR TS=(Depressions, Endogenous)) OR TS=(Endogenous Depression)) OR TS=(Endogenous Depressions)) OR TS=(Depressions, Neurotic)) OR TS=(Neurotic Depression)) OR TS=(Melancholia)) OR TS=(Melancholias)) OR TS=(Unipolar Depression)) OR TS=(affective disorders)) OR TS=(dysthymic disorder)) OR TS=(depressive disorder)) OR TS=(mood disorders) | 752593 |
| 2 | (((((((((((((TS=(Anxiety)) OR TS=(Anxiety Disorders)) OR TS=(Angst)) OR TS=(Social Anxiety)) OR TS=(Social Anxieties)) OR TS=(Hypervigilance)) OR TS=(Nervousness)) OR TS=(Anxiousness)) OR TS=(Anxiety Disorder)) OR TS=(Anxiety Neuroses)) OR TS=(Anxiety States, Neurotic)) OR TS=(Neurotic Anxiety State)) OR TS=(Neurotic Anxiety States)) OR TS=(State, Neurotic Anxiety) | 404324 |
| 3 | #1 AND #2 | 213552 |
| 4 | ((((((((TS=(Irritable Bowel Syndromes )) OR TS=(Irritable Bowel Syndrome)) OR TS=(Syndromes, Irritable Bowel )) OR TS=(Colon, Irritable)) OR TS=(Irritable Colon )) OR TS=(Colitis, Mucous)) OR TS=(Mucous Colitides)) OR TS=(Mucous Colitis)) OR TS=(IBS)) OR TS=(IBS-D)) | 32986 |
| 5 | ((((((((((((TS=(Traditional Chinese Medicine)) OR TS=(Zhong Yi Xue)) OR TS=(Chinese Traditional Medicine)) OR TS=(Chinese Medicine)) OR TS=(Traditional Tongue Diagnosis)) OR TS=(Tongue Diagnoses, Traditional)) OR TS=(Traditional Tongue Diagnoses)) OR TS=(Traditional Tongue Assessment)) OR TS=(herbal)) OR TS=(Traditional Chinese medicine compound)) OR TS=(herbal medicine)) OR TS=(Chinese herbal medicine)) | 117389 |
| 6 | (((AB=(randomized)) OR AB=(Randomized controlled trials)) OR AB=(RCT)) OR AB=(Clinical Control) | 1283594 |
| 7 | #3 AND #4 AND #5 AND #6 | 16 |

**Cochrane Library**

| **#** | **Search terms** | **Results** |
| --- | --- | --- |
| 1 | (Depression):ti,ab,kw or (Depressive Disorder):ti,ab,kw or (Depressive Symptoms):ti,ab,kw or (Depressive Symptomi):ti,ab,kw or (Emotional Depression):ti,ab,kw or (Symptom, Depressive):ti,ab,kw or (Depressive Disorders):ti,ab,kw or (Depressive Neuroses):ti,ab,kw or (Depressive Neurosis):ti,ab,kw or (Endogenous Depression):ti,ab,kw or (Endogenous Depressions):ti,ab,kw or (Depressions, Neurotic):ti,ab,kw or (Neurotic Depression):ti,ab,kw or (Melancholia):ti,ab,kw or (Melancholias):ti,ab,kw or (Unipolar Depression):ti,ab,kw or (affective disorders):ti,ab,kw or (dysthymic disorder):ti,ab,kw or (depressive disorder):ti,ab,kw or (mood disorders):ti,ab,kw or (Angst):ti,ab,kw | 114129 |
| 2 | (Anxiety):ti,ab,kw or (Anxiety Disorders):ti,ab,kw or (Social Anxiety):ti,ab,kw or (Social Anxieties):ti,ab,kw or (Hypervigilance):ti,ab,kw or (Nervousness):ti,ab,kw or (Anxiousness):ti,ab,kw or (Anxiety Disorder):ti,ab,kw or (Anxiety Neuroses):ti,ab,kw or (Neurotic Anxiety State):ti,ab,kw or (Anxiety States, Neurotic):ti,ab,kw or (Neurotic Anxiety State):ti,ab,kw or (Neurotic Anxiety States):ti,ab,kw | 77566 |
| 3 | #1 AND #2 | 42819 |
| 4 | (Irritable Bowel Syndrome):ti,ab,kw or (Irritable Bowel Syndromes):ti,ab,kw or (Syndromes, Irritable Bowel):ti,ab,kw or (Colon, Irritable):ti,ab,kw or (Irritable Colon ):ti,ab,kw or (Colitis, Mucous):ti,ab,kw or (Mucous Colitis):ti,ab,kw or (Mucous Colitides):ti,ab,kw or (IBS):ti,ab,kw or (IBS-D):ti,ab,kw | 5506 |
| 5 | (Traditional Chinese Medicine):ti,ab,kw or (Zhong Yi Xue):ti,ab,kw or (Chinese Traditional Medicine):ti,ab,kw or (Chinese Medicine):ti,ab,kw or (Traditional Tongue Diagnosis):ti,ab,kw or (Traditional Tongue Diagnoses):ti,ab,kw or (Traditional Tongue Assessment):ti,ab,kw or (Traditional Tongue Assessments):ti,ab,kw or (Traditional Chinese medicine compound):ti,ab,kw or (herbal):ti,ab,kw or (herbal medicine):ti,ab,kw or (Chinese herbal medicine):ti,ab,kw | 24926 |
| 6 | (randomized controlled trials):ti,ab,kw or (RCT):ti,ab,kw or (randomized):ti,ab,kw or (clinical control):ti,ab,kw or (placebo):ti,ab,kw or (clinical):ti,ab,kw or (Blind method):ti,ab,kw | 1545607 |
| 7 | #3 AND #4 AND #5 AND #6 | 43 |

**Embase via Ovid**

| **#** | **Search terms** | **Results** |
| --- | --- | --- |
| 1 | "Depression":ab,ti or "Depressive Disorder":ab,ti or "Depressive Symptoms":ab,ti or "Depressive Symptomi":ab,ti or "Emotional Depression":ab,ti or "Symptom, Depressive":ab,ti or "Depressive Disorders":ab,ti or "Depressive Neuroses":ab,ti or "Depressive Neurosis":ab,ti or "Depressions, Endogenous":ab,ti or "Endogenous Depression":ab,ti or "Endogenous Depressions":ab,ti or "Depressions, Neurotic":ab,ti or "Neurotic Depression":ab,ti or "Melancholia":ab,ti or "Melancholias":ab,ti or "Unipolar Depression":ab,ti or "dysthymic disorder":ab,ti or "affective disorders":ab,ti or "mood disorders":ab,ti or "depressive disorder":ab,ti or "Angst":ab,ti | 677085 |
| 2 | "Anxiety":ab,ti or "Anxiety Disorders":ab,ti or "Social Anxiety":ab,ti or "Social Anxieties":ab,ti or "Hypervigilance":ab,ti or "Nervousness":ab,ti or "Anxiousness":ab,ti or "Anxiety Disorder":ab,ti or "Anxiety Neuroses":ab,ti or "Neurotic Anxiety State":ab,ti or "Neurotic Anxiety State":ab,ti or "Anxiety States, Neurotic":ab,ti or "Neurotic Anxiety States":ab,ti or "State, Neurotic Anxiety":ab,ti | 393789 |
| 3 | #1 and #2 | 206011 |
| 4 | "Irritable Bowel Syndrome":ab,ti or "Irritable Bowel Syndromes":ab,ti or "Syndromes, Irritable Bowel":ab,ti or "Colon, Irritable":ab,ti or "Irritable Colon ":ab,ti or "Colitis, Mucous":ab,ti or "Mucous Colitides":ab,ti or "Mucous Colitis":ab,ti or "IBS":ab,ti or "IBS-D":ab,ti | 31549 |
| 5 | "Traditional Chinese Medicine":ab,ti or "Zhong Yi Xue":ab,ti or "Chinese Traditional Medicine":ab,ti or "Chinese Medicine":ab,ti or "Traditional Tongue Diagnosis":ab,ti or "Traditional Tongue Diagnoses":ab,ti or "Traditional Tongue Assessment":ab,ti or "Traditional Tongue Assessments":ab,ti or "Traditional Chinese medicine compound":ab,ti or "herbal medicine":ab,ti or "Chinese herbal medicine":ab,ti | 72873 |
| 6 | "RCT":ab,ti or "randomized":ab,ti or "clinical control":ab,ti or "placebo":ab,ti or "clinical":ab,ti or "randomized controlled trials":ab,ti | 7387861 |
| 7 | #3 and #4 and #5 and #6 | 14 |

**CNKI**

|  | **Search terms** | **Results** |
| --- | --- | --- |
| 1 | SU=抑郁 + 抑郁障碍 + 抑郁症 + 抑郁状态 AND SU=焦虑 + 焦虑状态 + 焦虑障碍 + 焦虑症 AND SU=中医 + 中药 + 方剂 + 中草药 + 中医药 + 中药汤剂 + 方药 + 复方 + 单方 AND SU=肠易激综合征 + IBS-D + 腹泻型肠易激综合征 AND AB=随机对照 + 随机 + RCT + 临床 + 对照 | 150 |

**Wanfang**

|  | **Search terms** | **Results** |
| --- | --- | --- |
| 1 | 主题:(抑郁 or 抑郁症 or 抑郁状态 or 抑郁障碍) and 主题:(焦虑障碍 or 焦虑 or 焦虑症 or 焦虑状态) and 主题:(肠易激综合征 or IBS or腹泻型肠易激综合征) and 主题:(中医 or 中药 or 方剂 or 方药 or 复方 or 单方 or 中医药 or 中草药 or 中药汤剂) and 摘要:(随机对照 or 随机 or 临床 or 对照 or RCT) | 269 |

**VIP**

|  | **Search terms** | **Results** |
| --- | --- | --- |
| 1 | (U=抑郁+U=抑郁症+U=抑郁状态+U=抑郁障碍) * (U=焦虑+U=焦虑状态+U=焦虑障碍+U=焦虑症)* (U=中医+ U=中药+ U=中医药+ U=方剂+ U=方药+U=中草药+ U=复方+U=单方+U=中药汤剂)*(U=肠易激综合征+U=IBS+U=腹泻型肠易激综合征）* (R=随机对照+ R=随机+ R=临床+ R=对照+ R=RCT) | 225 |

**CBM**

|  | **Search terms** | **Results** |
| --- | --- | --- |
| 1 | [("随机对照试验"[摘要:智能] OR "随机"[摘要:智能] OR "RCT"[摘要:智能] OR "对照"[摘要:智能] OR "临床"[摘要:智能]) AND (("中医"[常用字段:智能] OR "中药"[常用字段:智能] OR "中成药"[常用字段:智能] OR "方剂"[常用字段:智能] OR "方药"[常用字段:智能] OR "复方"[常用字段:智能] OR "单方"[常用字段:智能] OR "草药"[常用字段:智能] OR "汤药"[常用字段:智能]) OR ("肠易激综合征"[常用字段:智能] OR "肠易激"[常用字段:智能] OR "腹泻型肠易激综合征"[常用字段:智能] OR "IBS"[常用字段:智能])) AND ("肠易激综合征"[常用字段:智能] OR "肠易激"[常用字段:智能] OR "腹泻型肠易激综合征"[常用字段:智能] OR "IBS"[常用字段:智能]) AND ("焦虑"[常用字段:智能] OR "焦虑症"[常用字段:智能] OR "焦虑状态"[常用字段:智能] OR "焦虑障碍"[常用字段:智能]) AND ("抑郁"[常用字段:智能] OR "抑郁症"[常用字段:智能] OR "抑郁状态"[常用字段:智能] OR "抑郁障碍"[常用字段:智能])](javascript:toDoRelimitSearch();) | 757 |

**Table S2 Compositions of formulation and patented drugs**

| Study (year) | Formulation or  patented drugs | Source | Compositions | Quality control reported? |
| --- | --- | --- | --- | --- |
| Cai 2020 | Anchang decoction | **-** | *Bupleurum chinense DC.,* [Apiaceae;Bupleuri radix]30g, *Ligusticum chuanxiong Hort.,* [Apiaceae;Chuanxiong rhizoma]12g, *Citrus × aurantium L.,* [Rutaceae;Aurantii fructus]15g, *Atractylodes macrocephala Koidz.,* [Asteraceae;Atractylodis macrocephalae rhizoma]12g, *Citrus × aurantium L.,* [Rutaceae;Citri reticulatae pericarpium]15g, *Saposhnikovia divaricata (Turcz. ex Ledeb.) Schischk.,* [Apiaceae;Saposhnikoviae Radix]15g, *Cyperus rotundus L.,* [Cyperaceae;Cyperi rhizoma]12g, *Paeonia lactiflora Pall.,*[Paeoniaceae;Paeoniae radix alba]12g, *Glycyrrhiza uralensis Fisch. ex DC.,* [Fabaceae; Glycyrrhizae radix et rhizoma praeparata cum melle]6g. | N |
| Chen 2024 | Li Pi Qu Shi Fang | Guangdong Second Chinese Medicine Hospital | *Codonopsis pilosula (Franch.) Nannf. ,*[Campanulaceae;Codonopsis radix]20g*, Poria cocos (Schw.)Wolf .,*[Polyporaceae;Poria]10g*,Atractylodes lancea (Thunb.) DC.,* [Asteraceae;Atractylodis rhizoma]10g, *Atractylodes macrocephala Koidz. ,*[Asteraceae;Atractylodis macrocephalae rhizoma]10g*,Bupleurum chinense DC. ,*[Apiaceae;Bupleuri radix]10g,*Coix lacryma-jobi L.var.mayuen（Roman.）Stapf.,*[Poaceae;Coicis semen]20g*,Myristica fragrans Houtt.,* [Myristicaceae;Myristicae fragrantis aetheroleum]10g,*Saposhnikovia divaricata (Turcz. ex Ledeb.) Schischk.,* [Apiaceae;Radix saposhnikoviae]10g*,Paeonia lactiflora Pall. ,*[Paeoniaceae;Paeoniae radix alba]10g,*Citrus × aurantium L.,* [Rutaceae;Citri reticulatae pericarpium]10g*,Magnolia officinalis Rehder & E.H.Wilson.,* [Magnoliaceae;Cortex magnoliae officinalis]10g,*Corydalis yanhusuo (Y.H.Chou & Chun C.Hsu) W.T.Wang ex Z.Y.Su & C.Y.Wu.,* [Papaveraceae;Corydalis rhizoma]10g,*Glycyrrhiza uralensis Fisch. ex DC.,* [Fabaceae; Glycyrrhizae radix et rhizoma praeparata cum melle]6g. | N |
| Ding 2021 | Hepatogastric Dampening Tablets | Meizhou Academy of Medical Sciences | *Astragalus mongholicus Bunge.,* [Fabaceae;Astragali radix]30g, *Paeonia lactiflora Pall.,* [Paeoniaceae;Paeoniae radix alba]30g, *Poria cocos (Schw.)Wolf.,* [Polyporaceae;Poria]30g, *Atractylodes macrocephala Koidz.,* [Asteraceae;Atractylodis macrocephalae rhizoma]30g, *Raphanus raphanistrum subsp. sativus (L.) Domin.,* [Brassicaceae;Raphani semen]30g, *Citrus × aurantium L.,* [Rutaceae;Aurantii fructus immaturus]30g, *Atractylodes lancea (Thunb.) DC.,* [Asteraceae; Atractylodis rhizoma]30g*,*Massa Medicata Fermentata30g, *Agastache rugosa (Fisch. & C.A.Mey.) Kuntze.,* [Lamiaceae;Agastachis herba]60g*, Dolomiaea costus (Falc.) Kasana & A.K.Pandey.,* [Asteraceae;aucklandiae radix]15g. | N |
| Feng 2021 | Jian Pi Shen Shi Fang | Hubei Provincial Hospital of Chinese Medicine | *Codonopsis pilosula (Franch.) Nannf.,* [Campanulaceae;Codonopsis radix]10g, *Poria cocos (Schw.)Wolf.,*[Polyporaceae;Poria]10g, *Atractylodes macrocephala Koidz.,* [Asteraceae;Atractylodis macrocephalae rhizoma]15g, *Coix lacryma-jobi L.var.mayuen(Roman.)Stapf.,*[Poaceae;Coicis semen]20g, *Wurfbainia villosa (Lour.) Skornick. & A.D.Poulsen.,* [Zingiberaceae;Amomi fructus]6g, *Citrus × aurantium L.,*[Rutaceae;Citri reticulatae pericarpium]10g, *Dolomiaea costus (Falc.) Kasana & A.K.Pandey.,* [Asteraceae;aucklandiae radix]10g*, Prunus mume (Siebold) Siebold & Zucc.,* [Rosaceae;Fructus mume]15g,*Terminalia chebula Retz.,* [Combretaceae;Chebulae fructus]10g, *Punica granatum L.,* [Lythraceae;Cortex granati]15g, *Euryale ferox Salisb.,* [Nymphaeaceae;Euryales semen]10g, *Dioscorea oppositifolia L.,* [Dioscoreaceae;Dioscoreae rhizoma]15g, Massa Medicata Fermentata15g, *Atractylodes lancea (Thunb.) DC.,* [Asteraceae;Atractylodis rhizoma]10g, *Glycyrrhiza uralensis Fisch. ex DC.,* [Fabaceae; Glycyrrhizae radix et rhizoma praeparata cum melle]6g. | N |
| Fu 2013 | Shugan Lipi Zhixie decoction | Henan University of Chinese Medicine | *Bupleurum chinense DC.,* [Apiaceae;Bupleuri radix]12g, *Citrus × aurantium L.,* [Rutaceae;Citri reticulatae pericarpium]12g, *Atractylodes macrocephala Koidz.,* [Asteraceae;Atractylodis macrocephalae rhizoma]12g, *Paeonia lactiflora Pall.,*[Paeoniaceae;Paeoniae radix alba]30g, *Glycyrrhiza uralensis Fisch. ex DC.,* [Fabaceae; Glycyrrhizae radix et rhizoma praeparata cum melle]5g, *Saposhnikovia divaricata (Turcz. ex Ledeb.) Schischk.,* [Apiaceae;Saposhnikoviae Radix]10g, *Corydalis yanhusuo (Y.H.Chou & Chun C.Hsu) W.T.Wang ex Z.Y.Su & C.Y.Wu.,* [Papaveraceae;Corydalis rhizoma]15g, *Citrus × aurantium L.,* [Rutaceae;Aurantii fructus]10g, *Cyperus rotundus L.,* [Cyperaceae;Cyperi rhizoma]10g, *Crataegus pinnatifida Bunge.,* [Rosaceae;Crataegi fructus]15g, *Hordeum vulgare L.,* [Poaceae;Fructus hordei germinatus]20g, *Terminalia chebula Retz.*,[Combretaceae;Chebulae fructus]12g. | N |
| Gu 2022 | Baishi Wenpi decoction | Jiangsu Provincial Hospital of Chinese Medicine | *Atractylodes macrocephala Koidz.,* [Asteraceae;Atractylodis macrocephalae rhizoma]20g, *Acorus calamus var. angustatus Besser.,* [Acoraceae;Acori tatarinowii rhizoma]6g, *Glycyrrhiza uralensis Fisch. ex DC.,* [Fabaceae; Glycyrrhizae radix et rhizoma praeparata cum melle]6g, *Myristica fragrans Houtt.,* [Myristicaceae;Myristicae fragrantis aetheroleum]10g, *Dioscorea oppositifolia L.,* [Dioscoreaceae;Dioscoreae rhizoma]30g, *Coix lacryma-jobi L.var.mayuen(Roman）.Stapf.,*[Poaceae;Coicis semen]15g, *Agrimonia pilosa Ledeb.*, [Rosaceae;Agrimoniae herba]15g, *Euryale ferox Salisb.,*[Nymphaeaceae；Euryales semen]10g, *Prunus mume (Siebold) Siebold & Zucc.,* [Rosaceae;Fructus mume]6g, *Sanguisorba officinalis L.,* [Rosaceae;Radix sanguisorbae]15g, *Vincetoxicum mukdenense Kitag.,* [Apocynaceae;Cynanchi paniculati radix]15g, *Rosa rugosa Thunb.,* [Rosaceae;Flos rosae rugosae]6g. | N |
| Guo 2021 | Gu Chang Zhi Xie Wan | Shaanxi College of Traditional Chinese Medicine | *Prunus mume (Siebold) Siebold & Zucc.,*[Rosaceae;Fructus mume],*Coptis chinensis Franch.,* [Ranunculaceae;Coptidis rhizoma],*Dolomiaea costus (Falc.) Kasana & A.K.Pandey.,* [Asteraceae;aucklandiae radix],*Corydalis yanhusuo (Y.H.Chou & Chun C.Hsu) W.T.Wang ex Z.Y.Su & C.Y.Wu.,* [Papaveraceae;Corydalis rhizoma],*Dolomiaea costus (Falc.) Kasana & A.K.Pandey.,* [Asteraceae;aucklandiae radix],*Zingiber officinale Roscoe.,* [Zingiberaceae;Zingiberis Rhizoma Recens]. | N |
| He 2021 | Chaihu Guizhi Gangjiang decoction | Peili Nanning corporation | *Bupleurum chinense DC.,*[Apiaceae;Bupleuri radix]15g, *Trichosanthes kirilowii Maxim.*,[Cucurbitaceae;Fructus trichosanthis]25g, *Zingiber officinale Roscoe.,* [Zingiberaceae;Zingiberis rhizoma recens]10g, *Scutellaria baicalensis Georgi.,* [Lamiaceae;Scutellariae radix]20g, *Neolitsea cassia (L.) Kosterm.,* [Lauraceae;cinnamomi ramulus]15g, Ostreae concha]25g, *Glycyrrhiza uralensis Fisch. ex DC.,* [Fabaceae; Glycyrrhizae radix et rhizoma praeparata cum melle]10g. | N |
| Li 2021 | Shugan Hezhong decoction | Nanjing University of Chinese Medicine | *Bupleurum chinense DC.,* [Apiaceae;Bupleuri radix]10g, *Poria cocos (Schw.)Wolf.,*[Polyporaceae;Poria]10g, *Atractylodes macrocephala Koidz.,*[Asteraceae;Atractylodis macrocephalae rhizoma]10g, *Paeonia lactiflora Pall.,*[Paeoniaceae;Paeoniae radix alba]10g, *Angelica sinensis (Oliv.) Diels.,* [Apiaceae;Angelicae sinensis radix]10g, *Citrus × aurantium L.,* [Rutaceae;Citri reticulatae pericarpium]10g, *Saposhnikovia divaricata (Turcz. ex Ledeb.) Schischk.,* [Apiaceae;Saposhnikoviae Radix]10g, *Mentha canadensis L.,*[Lamiaceae;Menthae haplocalycis herba]3g, *Zingiber officinale Roscoe.,*[Zingiberaceae;Zingiberis rhizoma recens]3g, Os Draconis30g, Ostreae concha]30g*, Glycyrrhiza uralensis Fisch. ex DC.,* [Fabaceae; Glycyrrhizae radix et rhizoma praeparata cum melle]6g. | N |
| Liu1 2020 | Tongxie Lizhong decoction | North China University of Science and Technology | *Codonopsis pilosula (Franch.) Nannf.,* [Campanulaceae;Codonopsis radix]10g, *Paeonia lactiflora Pall.,* [Paeoniaceae;Paeoniae radix alba]10g, *Atractylodes macrocephala Koidz.,* [Asteraceae;Atractylodis macrocephalae rhizoma]10g, *Zingiber officinale Roscoe.,* [Zingiberaceae;Zingiberis rhizoma recens]7g, *Nelumbo nucifera Gaertn.*,[Nelumbonaceae;nelumbinis semen]15g, *Citrus × aurantium L.,* [Rutaceae;Citri reticulatae pericarpium]6g, *Saposhnikovia divaricata (Turcz. ex Ledeb.) Schischk.,* [Apiaceae;Saposhnikoviae Radix]6g, *Ligusticum chuanxiong Hort.,* [Apiaceae;Chuanxiong rhizoma]10g, *Plantago asiatica L.,* [Plantaginaceae;Herba plantaginis]10g, *Glycyrrhiza uralensis Fisch. ex DC.,*[Fabaceae; Glycyrrhizae radix et rhizoma praeparata cum melle]6g, Os Draconis15g, Ostreae Concha15g. | N |
| Liu2 2022 | Changning Tang Granules | Tanghe Hospital of Traditional Chinese Medicine | *Bupleurum chinense DC.,* [Apiaceae;Bupleuri radix]18g*,Paeonia lactiflora Pall.* [Paeoniaceae;Paeoniae radix alba]9g,*Atractylodes lancea (Thunb.) DC.,* [Asteraceae;Atractylodis rhizoma]12g,*Os Draconis24g,Codonopsis pilosula (Franch.) Nannf.,* [Campanulaceae;Codonopsis radix]9g,*Alisma plantago-aquatica subsp. orientale (Sam.) Sam.,* [Alismataceae;Alismatis rhizoma]15g,*Poria cocos (Schw.)Wolf.,* [Polyporaceae;Poria]15g,*Neolitsea cassia (L.) Kosterm.,* [Lauraceae;cinnamomi ramulus]6g,*Scutellaria baicalensis Georgi.,* [Lamiaceae;Scutellariae radix]6g,*Coptis chinensis Franch.,* [Ranunculaceae;Coptidis rhizoma]3g,*Glycyrrhiza uralensis Fisch. ex DC.,* [Fabaceae;Glycyrrhizae radix et rhizoma]6g. | N |
| Lu 2021 | Xiangsha Liujunzi Decoction |  | *Panax ginseng C.A.Mey.,* [Araliaceae;Ginseng radix et rhizoma]6g*,Pinellia ternata (Thunb.) Makino.,* [Araceae; Pinelliae rhizoma praeparatum cum alumine]6g,*Citrus × aurantium L.,* [Rutaceae;Citri reticulatae pericarpium]10g,*Atractylodes macrocephala Koidz.,* [Asteraceae;Atractylodis macrocephalae rhizoma]12g,*Poria cocos (Schw.)Wolf.,* [Polyporaceae;Poria]12g,*Wurfbainia villosa (Lour.) Skornick. & A.D.Poulsen.,* [Zingiberaceae;Amomi fructus]10g,*Dolomiaea costus (Falc.) Kasana & A.K.Pandey.,* [Asteraceae;aucklandiae radix]6g,*Glycyrrhiza uralensis Fisch. ex DC.,* [Fabaceae; Glycyrrhizae radix et rhizoma praeparata cum melle]10g. | N |
| Majing 2019 | Jieyu Tiaochang decoction | Shanxi University of Chinese Medicine | *Bupleurum chinense DC.,* [Apiaceae;Bupleuri radix]12g, *Angelica sinensis (Oliv.) Diels.,* [Apiaceae;Angelicae sinensis radix]12g, *Paeonia lactiflora Pall.,* [Paeoniaceae;Paeoniae radix alba]15g, *Atractylodes macrocephala Koidz.,* [Asteraceae;Atractylodis macrocephalae rhizoma]15g, *Citrus × aurantium L.,* [Rutaceae;Citri reticulatae pericarpium viride]12g, *Eleutherococcus senticosus (Rupr. & Maxim.) Maxim.,* [Araliaceae;Acanthopanacis senticosi radix et rhizoma seu caulis]12g, *Cinnamomum verum J.Presl.,* [Lauraceae;cinnamomi cortex]10g, *Albizia julibrissin Durazz.,* [Fabaceae;Albiziae flos]10g, *Rosa rugosa Thunb.,* [Rosaceae;Flos rosae rugosae]10g, *Citrus × aurantium f. aurantium.,*[Rutaceae;Aurantii fructus immaturus]10g, *Rhus chinensis Mill.,* [Anacardiaceae;Galla chinensis]5g. | N |
| Mou 2021 | Baishi Wenpi decoction | **-** | *Dioscorea oppositifolia L.,* [Dioscoreaceae;Dioscoreae rhizoma]30g, *Atractylodes macrocephala Koidz.,* [Asteraceae;Atractylodis macrocephalae rhizoma]20g, *Agrimonia pilosa Ledeb.,* [Rosaceae;Agrimoniae herba]15g, *Vincetoxicum mukdenense Kitag.,* [Apocynaceae;Cynanchi paniculati radix]15g, *Sanguisorba officinalis L.,* [Rosaceae;Radix sanguisorbae]15g, *Myristica fragrans Houtt.,* [Myristicaceae;Myristicae fragrantis aetheroleum]10g, *Euryale ferox Salisb.,* [Nymphaeaceae；Euryales semen]10g, *Zingiber officinale Roscoe.,* [Zingiberaceae;Zingiberis rhizoma recens]6g, *Prunus mume (Siebold) Siebold & Zucc.,* [Rosaceae;Fructus mume]6g, *Rosa rugosa Thunb.,* [Rosaceae;Flos rosae rugosae]6g, *Acorus calamus var. angustatus Besser.,* [Acoraceae;Acori tatarinowii rhizoma]6g. | N |
| Nie 2014 | Shugan Jianpi Compound Decoction | Dongfang Hospital of Beijing University of Chinese Medicine | *Cyperus rotundus L.,* [Cyperaceae;Cyperi rhizoma], *Paeonia lactiflora Pall.,* [Paeoniaceae;Paeoniae radix alba], *Atractylodes macrocephala Koidz.,* [Asteraceae;Atractylodis macrocephalae rhizoma], *Poria cocos (Schw.)Wolf.,*[Polyporaceae;Poria], *Prunus mume (Siebold) Siebold & Zucc.,* [Rosaceae;Fructus mume]. | N |
| Su 2022 | Peitu Shunmu Tang | Guangzhou University of Chinese Medicine | *Codonopsis pilosula (Franch.) Nannf.,* [Campanulaceae;Codonopsis radix]25g, *Atractylodes macrocephala Koidz.,* [Asteraceae;Atractylodis macrocephalae rhizoma]15g, *Dioscorea oppositifolia L.,* [Dioscoreaceae;Dioscoreae rhizoma]15g, *Poria cocos (Schw.)Wolf.,* [Polyporaceae;Poria]10g, *Citrus × aurantium L.,* [Rutaceae;Citri reticulatae pericarpium]5g, *Paeonia anomala subsp. veitchii (Lynch) D.Y.Hong & K.Y.Pan.,* [Paeoniaceae;Paeoniae radix rubra]10g, *Glycyrrhiza uralensis Fisch. ex DC.,* [Fabaceae; Glycyrrhizae radix et rhizoma praeparata cum melle]5g, *Bupleurum chinense DC.,* [Apiaceae;Bupleuri radix]5g, *Neolitsea cassia (L.) Kosterm.,* [Lauraceae;cinnamomi ramulus]5g, *Saposhnikovia divaricata (Turcz. ex Ledeb.) Schischk.,* [Apiaceae;Saposhnikoviae Radix]5g. | N |
| Sun 2020 | Jiawei Lichang | Shandong University of Chinese Medicine | *Atractylodes macrocephala Koidz.,* [Asteraceae;Atractylodis macrocephalae rhizoma]30g*, Poria cocos (Schw.)Wolf.,* [Polyporaceae;Poria]30g, *Paeonia lactiflora Pall.*,[Paeoniaceae;Paeoniae radix alba]15g*, Codonopsis pilosula (Franch.) Nannf.,* [Campanulaceae;Codonopsis radix]15g, *Bupleurum chinense DC.,* [Apiaceae;Bupleuri radix]12g, *Cyperus rotundus L.,* [Cyperaceae;Cyperi rhizoma]15g, *Alpinia officinarum Hance.,* [Zingiberaceae;Alpiniae officinarum rhizoma]6g, *Neolitsea cassia (L.) Kosterm.,* [Lauraceae;cinnamomi ramulus]9g, *Citrus × aurantium L.,* [Rutaceae;Citri reticulatae pericarpium]9g, *Saposhnikovia divaricata (Turcz. ex Ledeb.) Schischk.,* [Apiaceae;Saposhnikoviae Radix]9g, *Albizia julibrissin Durazz.,*[Fabaceae;Albiziae cortex]9g, *Glycyrrhiza uralensis Fisch. ex DC.,* [Fabaceae; Glycyrrhizae radix et rhizoma praeparata cum melle]6g. | N |
| Wu 2019 | Pingwei Capsules | Affiliated Hospital of Gansu University of TCM | *Atractylodes lancea (Thunb.) DC.,* [Asteraceae;Atractylodis rhizoma], *Citrus × aurantium L.,* [Rutaceae;Citri reticulatae pericarpium], *Citrus × aurantium L.,*[Rutaceae;Aurantii fructus], *Bupleurum chinense DC.,*[Apiaceae;Bupleuri radix], *Dolomiaea costus (Falc.) Kasana & A.K.Pandey.,* [Asteraceae;aucklandiae radix];*Sparganium stoloniferum (Buch.-Ham. ex Graebn.) Buch.-Ham. ex Juz.,* [Typhaceae;Sparganii rhizoma]; *Corydalis yanhusuo (Y.H.Chou & Chun C.Hsu) W.T.Wang ex Z.Y.Su & C.Y.Wu.*,[Papaveraceae;Corydalis rhizoma]; *Paeonia anomala subsp. veitchii (Lynch) D.Y.Hong & K.Y.Pan.,*[Paeoniaceae;Paeoniae radix rubra]. | N |
| Xu 2017 | Shugan Fupi Huashi Decoction | **-** | *Bupleurum chinense DC.,* [Apiaceae;Bupleuri radix]9g, *Paeonia lactiflora Pall.,*[Paeoniaceae;Paeoniae radix alba]20g,*Saposhnikovia divaricata (Turcz. ex Ledeb.) Schischk.,* [Apiaceae;Saposhnikoviae Radix]9g, *Atractylodes macrocephala Koidz.,* [Asteraceae;Atractylodis macrocephalae rhizoma]15g, *Citrus × aurantium L.,* [Rutaceae;Citri reticulatae pericarpium]15g, *Codonopsis pilosula (Franch.) Nannf.,* [Campanulaceae;Codonopsis radix]15g, *Poria cocos (Schw.)Wolf.,*[Polyporaceae;Poria]15g, *Prunus mume (Siebold) Siebold & Zucc.,* [Rosaceae;Fructus mume]15g, *Punica granatum L.,* [Lythraceae;Cortex granati]15g, *Pueraria montana var. lobata (Willd.) Maesen & S.M.Almeida ex Sanjappa & Predeep.,* [Fabaceae;Puerariae lobatae radix]15g*, Dolomiaea costus (Falc.) Kasana & A.K.Pandey.,*[Asteraceae;aucklandiae radix]6g, *Coptis chinensis Franch.,*[Ranunculaceae;Coptidis rhizoma]9g, *Glycyrrhiza uralensis Fisch. ex DC.,* [Fabaceae; Glycyrrhizae radix et rhizoma praeparata cum melle]9g. | N |
| Yang 2021 | Jiawei Jiaotai Wan | **-** | *Coptis chinensis Franch.,* [Ranunculaceae;Coptidis rhizoma]3g, *Cinnamomum verum J.Presl.,* [Lauraceae;cinnamomi cortex]3g, *Bupleurum chinense DC.,* [Apiaceae;Bupleuri radix]6g, *Atractylodes macrocephala Koidz.,* [Asteraceae;Atractylodis macrocephalae rhizoma]10g, *Paeonia lactiflora Pall.*,[Paeoniaceae;Paeoniae radix alba]20g, *Citrus × aurantium L.,* [Rutaceae;Citri reticulatae pericarpium]10g, *Saposhnikovia divaricata (Turcz. ex Ledeb.) Schischk.,* [Apiaceae;Saposhnikoviae Radix]10g, *Poria cocos (Schw.)Wolf.,* [Polyporaceae;Poria]15g, *Dioscorea oppositifolia L.,* [Dioscoreaceae;Dioscoreae rhizoma]20g, *Myristica fragrans Houtt.,* [Myristicaceae;Myristicae fragrantis aetheroleum]5g, *Zingiber officinale Roscoe.,* [Zingiberaceae;Zingiberis rhizoma recens]5g, *Dolomiaea costus (Falc.) Kasana & A.K.Pandey.,* [Asteraceae;aucklandiae radix]6g*, Schisandra chinensis (Turcz.) Baill.,* [Schisandraceae;Schisandrae chinensis fructus]10g, *Glycyrrhiza uralensis Fisch. ex DC.,* [Fabaceae; Glycyrrhizae radix et rhizoma praeparata cum melle]3g. | N |
| Zhang1 2016 | Modified Danggui Shaoyao Powder | Shijiazhuang Hospital of TCM | *Angelica sinensis (Oliv.) Diels.,* [Apiaceae;Angelicae sinensis radix]10g*, Paeonia lactiflora Pall.,* [Paeoniaceae;Paeoniae radix alba]15g, *Poria cocos (Schw.)Wolf.*, [Polyporaceae;Poria]12g*, Atractylodes macrocephala Koidz.,* [Asteraceae;Atractylodis macrocephalae rhizoma]12g, *Alisma plantago-aquatica subsp. orientale (Sam.) Sam.,*[Alismataceae;Alismatis rhizoma]10g, *Ligusticum chuanxiong Hort.,* [Apiaceae;Chuanxiong rhizoma]6g, *Glycyrrhiza uralensis Fisch. ex DC.,* [Fabaceae; Glycyrrhizae radix et rhizoma praeparata cum melle]6g, *Euryale ferox Salisb.,* [Nymphaeaceae；Euryales semen]6g, *Pseudocydonia sinensis (Dum.Cours.) C.K.Schneid.,* [Rosaceae;Chaenomelis fructus]10g. | N |
| Zhang2 2023 | Tongxie Yaofang and Wandai decoction | - | *Atractylodes macrocephala Koidz.,* [Asteraceae;Atractylodis macrocephalae rhizoma]15g,*Dioscorea oppositifolia L.,* [Dioscoreaceae;Dioscoreae rhizoma]15g,*Paeonia lactiflora Pall.,* [Paeoniaceae;Paeoniae radix alba]15g,*Panax ginseng C.A.Mey.,*[Araliaceae;Ginseng radix et rhizoma]10g,*Atractylodes lancea (Thunb.) DC.,* [Asteraceae;Atractylodis rhizoma]15g,*Plantago asiatica L.,* [Plantaginaceae;Herba plantaginis]15g,*Citrus × aurantium L.,* [Rutaceae;Citri reticulatae pericarpium]10g*,Saposhnikovia divaricata (Turcz. ex Ledeb.) Schischk.,* [Apiaceae;Radix saposhnikoviae]10g,*Bupleurum chinense DC. ,*[Apiaceae;Bupleuri radix]10g,*Glycyrrhiza uralensis Fisch. ex DC.,*[Fabaceae; Glycyrrhizae radix et rhizoma praeparata cum melle]6g. | N |
| Zhou 2020 | Modified Tongxie Yaofang | **-** | *Atractylodes macrocephala Koidz.,* [Asteraceae;Atractylodis macrocephalae rhizoma]30g, *Codonopsis pilosula (Franch.) Nannf.,* [Campanulaceae;Codonopsis radix]30g, *Paeonia lactiflora Pall.,* [Paeoniaceae;Paeoniae radix alba]20g, *Citrus × aurantium L.,* [Rutaceae;Citri reticulatae pericarpium]10g, *Dioscorea oppositifolia L.,* [Dioscoreaceae;Dioscoreae rhizoma]15g, *Glycyrrhiza uralensis Fisch. ex DC.,*[Fabaceae; Glycyrrhizae radix et rhizoma praeparata cum melle]15g, *Poria cocos (Schw.)Wolf.,*[Polyporaceae;Poria]15g, *Citrus × aurantium L.,*[Rutaceae;Aurantii fructus]12g, *Saposhnikovia divaricata (Turcz. ex Ledeb.) Schischk.,* [Apiaceae;Saposhnikoviae Radix]10g, *Curcuma aromatica Salisb.,*[Zingiberaceae;Curcumae radix]10g, *Coptis chinensis Franch.,* [Ranunculaceae;Coptidis rhizoma]6g. | N |
| Zhu1 2013 | Shugan Lipi Fang | **-** | *Atractylodes macrocephala Koidz.,*[Asteraceae;Atractylodis macrocephalae rhizoma]15g, *Paeonia lactiflora Pall.,*[Paeoniaceae;Paeoniae radix alba]12g, *Citrus × aurantium L.,* [Rutaceae;Citri reticulatae pericarpium]9g, *Poria cocos (Schw.)Wolf.,* [Polyporaceae;Poria]9g, *Bupleurum chinense DC.,* [Apiaceae;Bupleuri radix]9g, *Actaea cimicifuga L.,*[Ranunculaceae;Cimicifugae rhizoma]6g, *Saposhnikovia divaricata (Turcz. ex Ledeb.) Schischk.,* [Apiaceae;Saposhnikoviae Radix]6g. | N |
| Zhu2  2015 | Chaihu Shugan San and Tongxie Yaofang | Xi'an Janssen Pharmaceutical Company Limited | *Bupleurum chinense DC.,* [Apiaceae;Bupleuri radix]10g,*Atractylodes macrocephala Koidz.,* [Asteraceae;Atractylodis macrocephalae rhizoma]10g,*Paeonia lactiflora Pall.,* [Paeoniaceae;Paeoniae radix alba]15g,*Saposhnikovia divaricata (Turcz. ex Ledeb.) Schischk.,* [Apiaceae;Radix saposhnikoviae]6g,*Citrus × aurantium L.,* [Rutaceae;Citri reticulatae pericarpium]10g,*Cyperus rotundus L.,* [Cyperaceae;Cyperi rhizoma]10g,*Ligusticum chuanxiong Hort.,* [Apiaceae;Chuanxiong rhizoma]6g,*Poria cocos (Schw.)Wolf.,* [Polyporaceae;Poria]15g,*Dioscorea oppositifolia L.,*[Dioscoreaceae;Dioscoreae rhizoma]20g,*Wurfbainia villosa (Lour.) Skornick. & A.D.Poulsen.,* [Zingiberaceae;Amomi fructus]15g,*Glycyrrhiza uralensis Fisch. ex DC.,* [Fabaceae; Glycyrrhizae radix et rhizoma praeparata cum melle]6g. |  |

**Table S3 The frequency statistics of CHM.**

| Local name | English name | Latin name | Frequency |
| --- | --- | --- | --- |
| Baizhu | Atractylodis macrocephalae rhizoma | *Atractylodes macrocephala Koidz.* | 20 |
| Baishao | Paeoniae radix  alba | *Paeonia lactiflora Pall.* | 17 |
| Zhigancao | Glycyrrhizae Radix Et Rhizoma Praeparata Melle | *Glycyrrhiza uralensis Fisch. ex DC.* | 16 |
| Chenpi | Citri reticulatae pericarpium | *Citrus × aurantium L.* | 16 |
| Fuling | Poria | *Poria Cocos(Schw.) Wolf.* | 15 |
| Chaihu | Bupleuri radix | *Bupleurum chinense DC.* | 15 |
| Fangfeng | Saposhnikoviae Radix | *Saposhnikovia divaricata (Turcz.) Schischk* | 13 |
| Shanyao | Dioscorea opposita Thunb | *Dioscorea oppositifolia L.* | 8 |
| Dangshen | Codonopsis radix | *Codonopsis pilosula (Franch.)*  *Nannf.* | 8 |
| Muxiang | Aucklandiae radix | *Dolomiaea costus (Falc.) Kasana & A.K.Pandey.* | 7 |
| Wumei | Fructus mume | *Prunus mume (Siebold) Siebold & Zucc.* | 6 |
| Cangzhu | Atractylodes Rhizome | *Atractylodes Lancea (Thunb.) DC.* | 6 |
| Ganjiang | Zingiberis Rhizoma Recens | *Zingiber Officinale Roscoe* | 5 |
| Huanglian | Coptidis rhizoma | *Coptis chinensis Franch.* | 5 |
| Xiangfu | Cyperi rhizoma | *Cyperus rotundus L.* | 5 |
| Zhiqiao | Aurantii fructus | *Citrus×aurantium L.* | 4 |
| Yiyiren | Coicis semen | *Coix lacryma-jobi L.var.mayuen（Roman.）Stapf.* | 4 |
| Yanhusuo | Corydalis Rhizoma | *Corydalis yanhusuo (Y.H.Chou & Chun C.Hsu) W.T.Wang ex Z.Y.Su & C.Y.Wu* | 4 |
| Chuanxiong | Chuanxiong rhizoma | *Ligusticum chuanxiong Hort.* | 4 |
| Qianshi | Euryales semen | *Euryale ferox Salisb.* | 4 |
| Guizhi | Cinnamomi Ramulus | *Neolitsea cassia (L.) Kosterm.* | 4 |
| Danggui | Angelicae sinensis  radix | *Angelica sinensis (Oliv.) Diels* | 3 |
| Meiguihua | Flos rosae rugosae | *Rosa rugosa Thunb.* | 3 |
| Longgu | Os Draconis |  | 3 |
| Muli | Ostreae Concha |  | 3 |
| Roudoukou | Myristicae fragrantis aetheroleum | *Myristica fragrans Houtt.* | 3 |

**Table S4 Subgroup analysis comparing HAMD scores of the trial group and the control group after treatment**

| Studies characteristics | Subgroup | Number of studies included | Heterogeneity test results | | Meta analysis results | |
| --- | --- | --- | --- | --- | --- | --- |
|  |  |  | I^2^ | *P* | SMD(95%CI) | *P* |
| TCM syndrome | liver depression and spleen deficiency | 13 | 71 | *P*＜0.0001 | -1.09 [-1.34, -0.84] | *P*＜0.00001 |
|  | other syndrome types | 2 | 31 | *P*=0.23 | -1.01 [-1.45, -0.56] | *P*＜0.00001 |
| Intervention time | ≤ 4 weeks | 9 | 7 | *P*=0.38 | -0.98 [-1.16, -0.80] | *P*＜0.00001 |
|  | ＞4 weeks | 6 | 85 | *P*＜0.00001 | -1.21 [-1.67, -0.74] | *P*＜0.00001 |
| Interventions | CHM vs Western medicine | 11 | 67 | *P*=0.0007 | -1.11 [-1.37, -0.85] | *P*＜0.00001 |
|  | CHM+Western medicine vs Western medicine | 4 | 73 | *P*=0.01 | -0.98 [-1.42, -0.54] | *P*＜0.0001 |

**Table S5 Subgroup analysis comparing HAMA scores of the trial group and the control group after treatment**

| Studies characteristics | Subgroup | Number of studies included | Heterogeneity test results | | Meta analysis results | |
| --- | --- | --- | --- | --- | --- | --- |
|  |  |  | I^2^ | *P* | SMD(95%CI) | *P* |
| TCM syndrome | liver depression and spleen deficiency | 13 | 90 | *P*＜0.00001 | -1.36 [-1.80, -0.93] | *P*＜0.00001 |
|  | other syndrome types | 2 | 37 | *P*=0.21 | -0.89 [-1.35, -0.43] | *P*=0.0002 |
| Intervention time | ≤ 4 weeks | 9 | 89 | *P*＜0.00001 | -1.35 [-1.89, -0.80] | *P*＜0.00001 |
|  | ＞4 weeks | 6 | 91 | *P*＜0.00001 | -1.24 [-1.84, -0.63] | *P*＜0.0001 |
| Interventions | CHM vs Western medicine | 11 | 91 | *P*＜0.00001 | -1.44 [-1.97, -0.91] | *P*＜0.00001 |
|  | CHM+Western medicine vs Western medicine | 4 | 50 | *P*=0.11 | -0.95 [-1.28, -0.63] | *P*＜0.00001 |

**Table S6 Subgroup analysis comparing SDS scores of the trial group and the control group after treatment**

| Studies characteristics | Subgroup | Number of studies included | Heterogeneity test results | | Meta analysis results | |
| --- | --- | --- | --- | --- | --- | --- |
|  |  |  | I^2^ | *P* | SMD(95%CI) | *P* |
| TCM syndrome | liver depression and spleen deficiency | 6 | 12 | *P*=0.34 | -0.58 [-0.76, -0.40] | *P*＜0.00001 |
|  | other syndrome types | 4 | 98 | *P*＜0.00001 | -4.01 [-6.46, -1.56] | *P*=0.001 |
| Intervention time | ≤ 4 weeks | 8 | 97 | *P*＜0.00001 | -2.12 [-3.16, -1.08] | *P*＜0.0001 |
|  | ＞4 weeks | 2 | 37 | *P*=0.21 | -0.49 [-0.77, -0.21] | *P*=0.0007 |
| Interventions | CHM vs Western medicine | 6 | 30 | *P*=0.21 | -0.51 [-0.71, -0.32] | *P*＜0.00001 |
|  | CHM+Western medicine vs Western medicine | 4 | 98 | *P*＜0.00001 | -4.03 [-6.17, -1.89] | *P*=0.0002 |

**Table S7 Subgroup analysis comparing SAS scores of the trial group and the control group after treatment**

| Studies characteristics | Subgroup | Number of studies included | Heterogeneity test results | | Meta analysis results | |
| --- | --- | --- | --- | --- | --- | --- |
|  |  |  | I^2^ | *P* | SMD(95%CI) | *P* |
| TCM syndrome | liver depression and spleen deficiency | 6 | 82 | *P*＜0.0001 | -2.18 [-3.21, -1.15] | *P*=0.005 |
|  | other syndrome types | 4 | 98 | *P*＜0.00001 | -3.77 [-5.80, -1.74] | *P*=0.0003 |
| Intervention time | ≤ 4 weeks | 8 | 97 | *P*＜0.00001 | -2.18 [-3.21, -1.15] | *P*＜0.0001 |
|  | ＞4 weeks | 2 | 89 | *P*=0.002 | -0.37 [-1.30, 0.57] | *P*=0.45 |
| Interventions | CHM vs Western medicine | 6 | 82 | *P*＜0.0001 | -0.73 [-1.17, -0.28] | *P*=0.002 |
|  | CHM+Western medicine vs Western medicine | 4 | 98 | *P*＜0.00001 | -3.75 [-6.09, -1.42] | *P*=0.002 |

**Table S8 Subgroup analysis comparing IBS-SSS scores of the trial group and the control group after treatment**

| Studies characteristics | Subgroup | Number of studies included | Heterogeneity test results | | Meta analysis results | |
| --- | --- | --- | --- | --- | --- | --- |
|  |  |  | I^2^ | *P* | SMD(95%CI) | *P* |
| TCM syndrome | liver depression and spleen deficiency | 9 | 84 | *P*＜0.00001 | -1.18 [-1.61, -0.75] | *P*＜0.00001 |
|  | other syndrome types | 2 | 0 | *P*=0.64 | -1.55 [-1.91, -1.19] | *P*＜0.00001 |
| Intervention time | ≤ 4 weeks | 10 | 84 | *P*＜0.00001 | -1.25 [-1.66, -0.83] | *P*＜0.00001 |
|  | ＞4 weeks | 1 | Not Applicable | Not Applicable | -1.25 [-1.61, -0.88] | *P*＜0.00001 |
| Interventions | CHM vs Western medicine | 8 | 85 | *P*＜0.00001 | -1.19 [-1.68, -0.71] | *P*＜0.00001 |
|  | CHM+Western medicine vs Western medicine | 3 | 48 | *P*=0.15 | -1.42 [-1.69, -1.14] | *P*＜0.00001 |

**Table S9 Subgroup analysis of the comparison of TCM symptom scores between the trial group and the control group after treatment**

| Studies characteristics | Subgroup | Number of studies included | Heterogeneity test results | | Meta analysis results | |
| --- | --- | --- | --- | --- | --- | --- |
|  |  |  | I^2^ | *P* | SMD(95%CI) | *P* |
| TCM syndrome | liver depression and spleen deficiency | 12 | 94 | *P*＜0.00001 | -1.69 [-2.32, -1.06] | *P*＜0.00001 |
|  | other syndrome types | 5 | 96 | *P*＜0.00001 | -2.26 [-3.67, -0.84] | *P*=0.002 |
| Intervention time | ≤ 4 weeks | 13 | 95 | *P*＜0.00001 | -2.10 [-2.82, -1.38] | *P*＜0.00001 |
|  | ＞4 weeks | 4 | 95 | *P*＜0.00001 | -1.40 [-2.50, -0.29] | *P*=0.01 |
| Interventions | CHM vs Western medicine | 12 | 89 | *P*＜0.00001 | -1.23 [-1.71, -0.71] | *P*＜0.00001 |
|  | CHM+Western medicine vs Western medicine | 5 | 95 | *P*＜0.00001 | -3.01 [-4.24, -1.78] | *P*＜0.00001 |

**
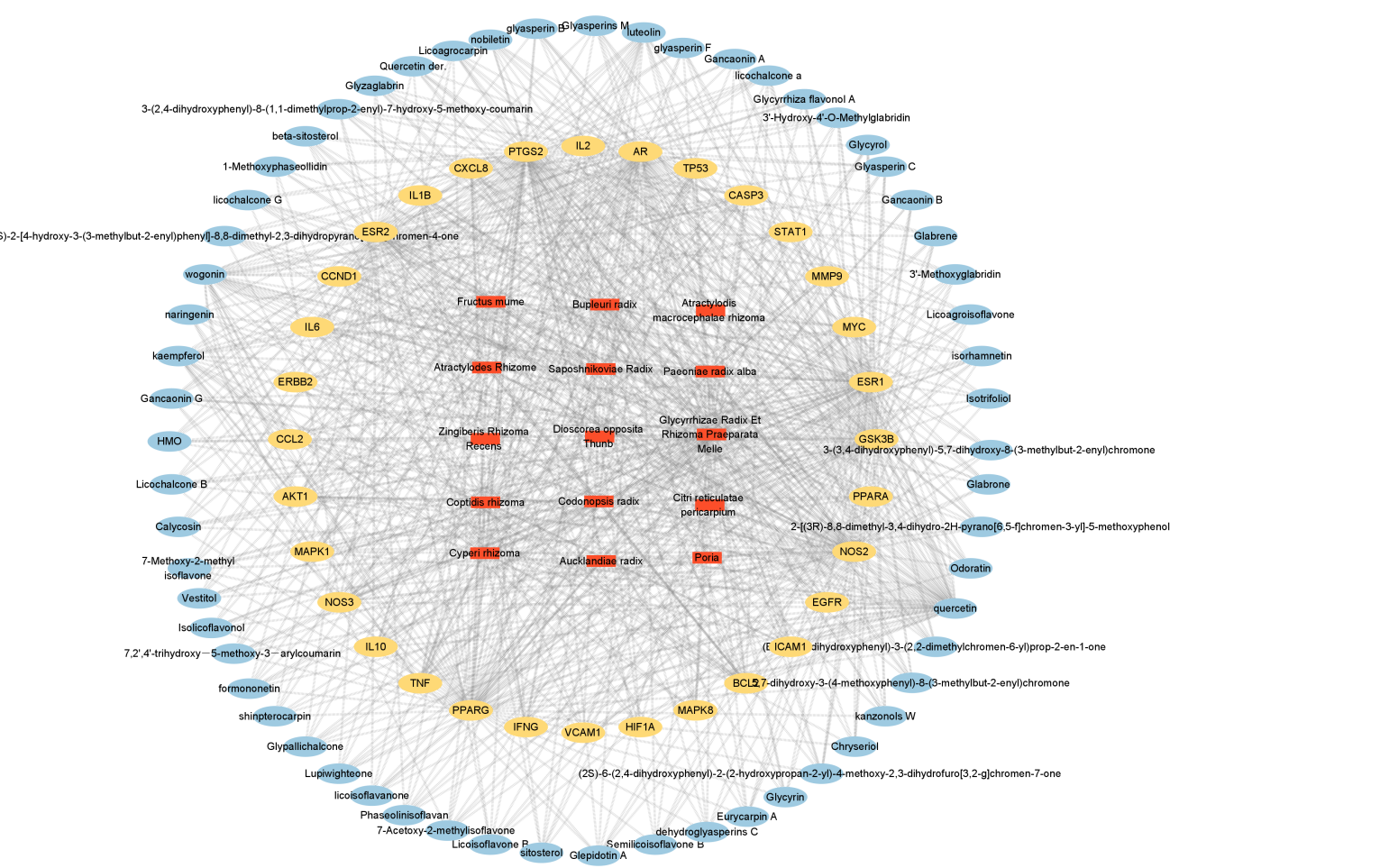
**

Figure S1. The networks of active ingredients-disease targets of IBS-D with depression and anxiety. Red: CHM;yellow: disease targets; blue: active ingredients.
